# Innate immune adaptor TRIF deficiency accelerates disease progression of ALS mice with accumulation of aberrantly activated astrocytes

**DOI:** 10.1038/s41418-018-0098-3

**Published:** 2018-03-22

**Authors:** Okiru Komine, Hirofumi Yamashita, Noriko Fujimori-Tonou, Masato Koike, Shijie Jin, Yasuhiro Moriwaki, Fumito Endo, Seiji Watanabe, Satoshi Uematsu, Shizuo Akira, Yasuo Uchiyama, Ryosuke Takahashi, Hidemi Misawa, Koji Yamanaka

**Affiliations:** 1 Department of Neuroscience and Pathobiology, Research Institute of Environmental Medicine, Nagoya University, Nagoya, Japan; 2 Laboratory for Motor Neuron Disease, RIKEN Brain Science Institute, Wako, Japan; 3 Department of Neurology, Graduate School of Medicine, Kyoto University, Kyoto, Japan; 4 Laboratory for Molecular Dynamics of Mental Disorders, RIKEN Brain Science Institute, Wako, Japan; 5 Department of Cell Biology and Neuroscience, Juntendo University Graduate School of Medicine, Tokyo, Japan; 6 Division of Pharmacology, Faculty of Pharmacy, Keio University, Tokyo, Japan; 7 Department of Mucosal Immunology, School of Medicine, Chiba University, Chiba, Japan; 8 Division of Innate Immune Regulation, International Research and Development Center for Mucosal Vaccines, Institute of Medical Science, The University of Tokyo, Tokyo, Japan; 9 Laboratory of Host Defense, World Premier International Immunology Frontier Research Center, and Department of Host Defense, Research Institute for Microbial Diseases, Osaka University, Osaka, Japan; 10 Department of Cellular and Molecular Neuropathology, Juntendo University Graduate School of Medicine, Tokyo, Japan; 11 Department of Neuroscience and Pathobiology, Nagoya University Graduate School of Medicine, Nagoya, Japan

## Abstract

There is compelling evidence that glial-immune interactions contribute to the progression of neurodegenerative diseases. The adaptive immune response has been implicated in disease processes of amyotrophic lateral sclerosis (ALS), but it remains unknown if innate immune signaling also contributes to ALS progression. Here we report that deficiency of the innate immune adaptor TIR domain-containing adaptor inducing interferon-β (TRIF), which is essential for certain Toll-like receptor (TLR) signaling cascades, significantly shortens survival time and accelerates disease progression of ALS mice. While myeloid differentiation factor 88 (MyD88) is also a crucial adaptor for most TLR signaling pathways, MyD88 deficiency had only a marginal impact on disease course. Moreover, TRIF deficiency reduced the number of natural killer (NK), NK-T-lymphocytes, and CD8-T cells infiltrating into the spinal cord of ALS mice, but experimental modulation of these populations did not substantially influence survival time. Instead, we found that aberrantly activated astrocytes expressing Mac2, p62, and apoptotic markers were accumulated in the lesions of TRIF-deficient ALS mice, and that the number of aberrantly activated astrocytes was negatively correlated with survival time. These findings suggest that TRIF pathway plays an important role in protecting a microenvironment surrounding motor neurons by eliminating aberrantly activated astrocytes.

## Introduction

Accumulating evidence implicates the immune dysfunction and neuroinflammation in the progression of etiologically distinct neurodegenerative diseases, [1–4] including amyotrophic lateral sclerosis (ALS), an adult onset neurodegenerative disease characterized by selective loss of motor neurons. About 10% of ALS cases are inherited, and a dominant mutation in the gene for Cu/Zn superoxide dismutase (SOD1) accounts for ≈20% of all familial cases.

One of the common pathological findings in ALS and other neurodegenerative diseases is neuroinflammation involving activated glial cells, such as microglia and astrocytes, along with infiltrating T-lymphocytes. These non-neuronal elements affect the fate of motor neurons through a “non-cell autonomous” mechanism [5–7]. Our previous works and those of others demonstrated that selective reduction of mutant SOD1 expression in microglia [8–10], astrocytes [11, 12], or oligodendrocytes [13] significantly slows the disease progression of mutant SOD1-ALS mice. In contrast, elimination of functional T-lymphocytes or CD4^+^ T-lymphocytes from mutant SOD1 mice was reported to further shorten survival [14, 15]. While the contributions of acquired immunity, such as effects mediated by T-lymphocytes, have been extensively investigated in ALS mice [16, 17], the functions of innate immune signaling pathways in ALS are still largely unknown.

The innate immune system is the first line of defense for protecting the host from invading pathogens. Microglia are considered as the central mediators of the innate immune response in the central nervous system (CNS); however, previous reports revealed that astrocytes and oligodendrocytes also express innate immune receptors and initiate innate immune responses [18, 19]. The Toll-like receptor (TLR) family plays a key role in innate immune responses by recognizing pathogen-associated molecular patterns and damage-associated molecular patterns. These TLR-mediated responses require myeloid differentiation factor 88 (MyD88) and (or) TIR domain-containing adaptor inducing interferon-β (TRIF) as essential adaptor proteins [20]. All TLR signaling pathways except that induced by TLR3 are dependent on MyD88, while TRIF is required for TLR3-mediated signaling and TLR4 activates both MyD88-associated and TRIF-associated pathways. These TLR pathways trigger the production of various pro-inflammatory cytokines, chemokines, and type I interferons through activation of transcription factors nuclear factor-κB (NF-κB), AP-1, IRF3, and IRF7 to eliminate pathogens and viruses [20]. Unlike MyD88-dependent pathways, TRIF-dependent TLR3/4 pathways are also able to eliminate host cells by inducing apoptosis through caspase-8 activation, thereby inhibiting viral propagation [21].

TLRs also recognize abnormal proteins linked to neurodegenerative diseases, triggering inflammatory responses in the CNS [22]. For example, TLR2, TLR4, and their co-receptor CD14 are involved in the recognition and clearance of amyloid-β in the mouse models of Alzheimer’s disease [4]. A previous study showed that bone marrow deficiency of MyD88 accelerates disease progression in chimeric SOD1^G37R^ mice, implicating TLR signaling in ALS [23]. However, MyD88-null SOD1^G37R^ mice exhibited no change in disease onset or survival times [23]. Similarly, deficiency of CD14 had no effect on the survival time of SOD1^G93A^ mice [24]. On the other hand, TLR4 deficiency prolonged the survival of SOD1^G93A^ mice [25]. Since TLR4 activates both MyD88-dependent and TRIF-dependent signaling pathways, the individual contributions of these pathways remain unclear.

Activation of microglia and astrocytes is a key process in neuroinflammation, and persistent neuroinflammation driven by these cells is detrimental to the cellular environment in the CNS, thereby exacerbating neurodegeneration. However, it remains unknown how the neuroinflammation is controlled and terminated or how overactivated glial cells are eliminated in neurodegenerative diseases.

In this study, we re-evaluate the role of innate immune pathways in ALS and revealed that TRIF-dependent signaling, but not MyD88-dependent signaling, is crucial for disease progression in SOD1^G93A^ mice. We also found that aberrantly activated astrocytes in addition to activated microglia accumulated in TRIF-deficient SOD1^G93A^ spinal cord starting at disease onset. Moreover, a negative correlation was observed between the number of aberrantly activated astrocytes and the survival time of TRIF-deficient SOD1^G93A^ mice. Collectively, these results revealed for the first time that the TRIF pathway is involved in eliminating aberrantly activated astrocytes to control neuroinflammation and maintain the microenvironment surrounding motor neurons in ALS mice.

## Results

### Genetic deletion of TRIF in SOD1^G93A^ mice significantly shortens life span by accelerating disease progression

**Fig. 1:**
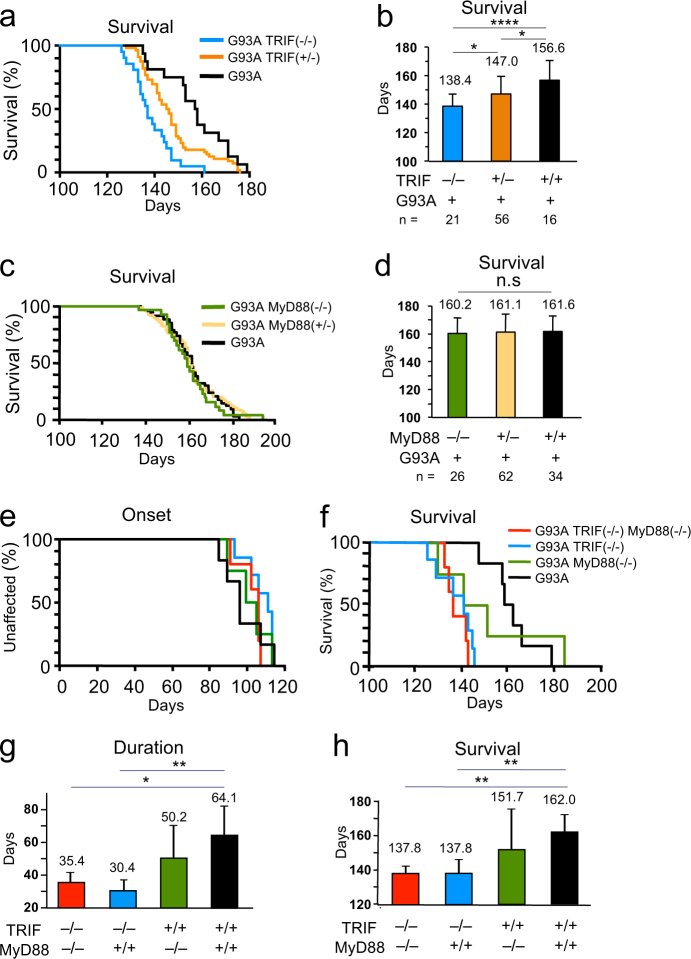
Genetic deletion of TRIF significantly shortens the life span of SOD1^G93A^ mice by accelerated disease progression. **a** Survival curves of SOD1^G93A^ (black, *n* = 16), SOD1^G93A^/TRIF^+/−^ (orange, *n* = 56), and SOD1^G93A^/TRIF^−/−^ (light blue, *n* = 21) mice produced by a double mating of SOD1^G93A^ and TRIF^−/−^ mice. **b** Plotted mean survival time of each genotype showed that heterozygous and homozygous deletion of *TRIF* gene shortened survival times of SOD1^G93A^ mice. Data are presented as mean ± SD. **c** Survival curve of SOD1^G93A^ (black, *n* = 34), SOD1^G93A^/MyD88^+/−^ (yellow, *n* = 62), and are SOD1^G93A^/MyD88^−/−^ (green, *n* = 26) mice produced by a double mating of SOD1^G93A^ and MyD88^−/−^ mice. **d** Plotted mean survival times showed that the survival times of SOD1^G93A^ mice were not affected by *MyD88* gene deletion. Data are presented as mean ± SD. **e**, **f** Ages of disease onset (**e**) and disease end stage (**f**) for SOD1^G93A^/TRIF^−/−^/MyD88^−/−^ (red, *n* = 5), SOD1^G93A^/TRIF^−/−^ (blue, *n* = 7), SOD1^G93A^/MyD88^−/−^ (green, *n* = 4), and littermates SOD1^G93A^ mice (black, *n* = 6) produced by a triple mating of SOD1^G93A^, TRIF^−/−^, and MyD88^−/−^ mice. **g** Mean duration of disease (days from onset to end stage) for SOD1^G93A^/TRIF^−/−^/MyD88^−/−^ (red), SOD1^G93A^/TRIF^−/−^ (blue), SOD1^G93A^/MyD88^−/−^ (green), and littermate SOD1^G93A^ mice (black). **h** Mean survival time of each genotype shown in **f**. Data are presented as mean ± SD. Statistical analyses of survival time (**b**, **d**, **h**) and disease duration (**g**) were performed with log-rank tests with Bonferroni corrections for multiple comparisons and one-way ANOVA followed by Tukey–Kramer multiple comparison post hoc tests, respectively. *p < 0.05, **p < 0.01, ****p < 0.0001

To investigate the role of the TLR-dependent innate immune signaling in the mouse models of ALS, we mated SOD1^G93A^ mice with mice deficient in MyD88 or TRIF, essential adaptor proteins for TLR signaling by double mating. We found that TRIF deficiency, but not MyD88 deficiency, substantially shortened the survival time of SOD1^G93A^ mice (SOD1^G93A^/TRIF^−/−^, 138.4 ± 8.6 days; SOD1^G93A^/TRIF^+/−^, 147.0 ± 12.4 days; SOD1^G93A^, 156.6 ± 13.8 d; Fig. 1a, b), (SOD1^G93A^/MyD88^−/−^, 160.2 ± 11.2 days; SOD1^G93A^/MyD88^+/−^, 161.1 ± 13.0 days; SOD1^G93A^, 161.6 ± 11.3 days; Fig. 1c, d). To exclude the influence of subtle genetic background, we also generated SOD1^G93A^/MyD88^−/−^/TRIF^−/−^, SOD1^G93A^/MyD88^−/−^, and SOD1^G93A^/TRIF^−/−^ mice, and littermate SOD1^G93A^ mice by multi-step triple mating at a probability of 3.1% (1/32) each and performed survival analyses. Disease onset was unaffected by ablation of MyD88 or TRIF from SOD1^G93A^ mice (SOD1^G93A^, 97.8 ± 10.9 days; SOD1^G93A^/MyD88^−/−^, 101.5 ± 10.1 days; SOD1^G93A^/TRIF^−/−^, 107.4 ± 7.7 days; SOD1^G93A^/MyD88^−/−^/TRIF^−/−^, 102.4 ± 6.6 days; Fig. 1e); however, disease progression was substantially accelerated when TRIF was eliminated from SOD1^G93A^ mice as evidenced by a 50% reduction in disease duration (SOD1^G93A^, 64.1 ± 17.8 days; SOD1^G93A^/MyD88^−/−^, 50.2 ± 19.9 days; SOD1^G93A^/TRIF^−/−^, 30.4 ± 5.8 days; SOD1^G93A^/MyD88^−/−^/TRIF^−/−^, 35.4 ± 6.9 days; Fig. 1g). Similar to results of the double mating experiments, overall survival time was shortened by 24 days when TRIF was deleted (SOD1^G93A^, 162.0 ± 10.2 days; SOD1^G93A^/MyD88^−/−^, 151.7 ± 23.7 days; SOD1^G93A^/TRIF^−/−^, 137.8 ± 8.1 days; SOD1^G93A^/MyD88^−/−^/TRIF^−/−^, 137.8 ± 4.4 days; Fig. 1f, h). In contrast, MyD88 deficiency alone did not affect disease onset, progression, or survival time of SOD1^G93A^ mice (Fig. 1c–h). This series of mating experiments indicates that the TRIF-dependent innate immune pathway determines disease progression in mutant SOD1 mice.

### Activation state of spinal cord microglia is similar in SOD1^G93A^ and SOD1^G93A^/TRIF^−/−^ mice at disease end stage

**Fig. 2:**
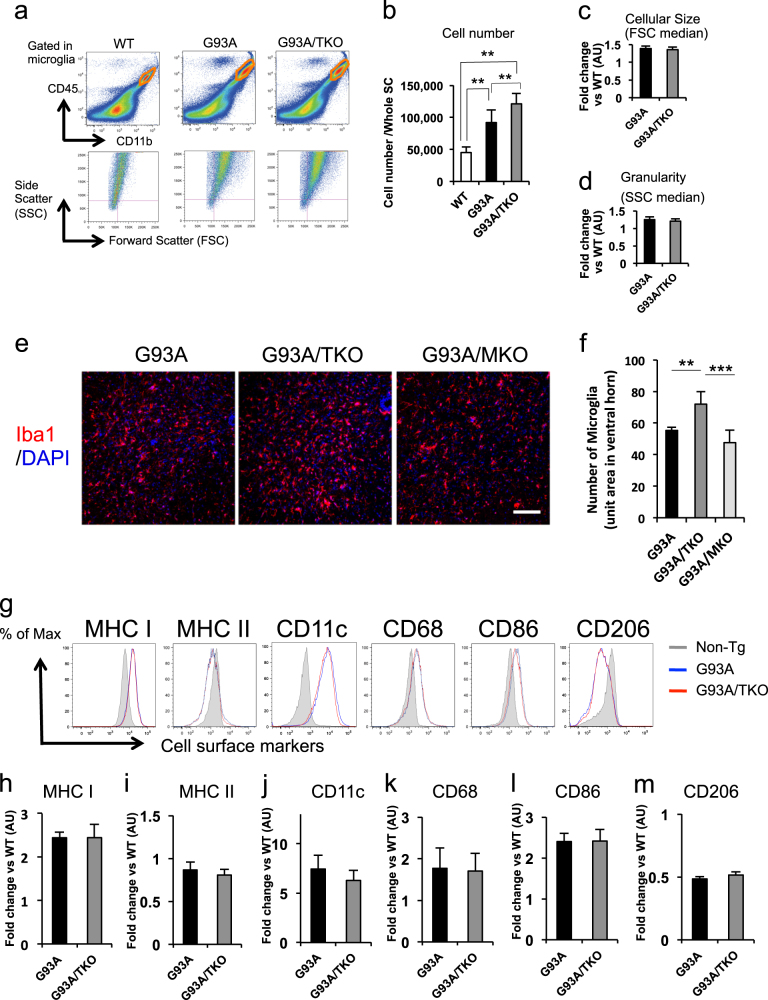
Deletion of TRIF in SOD1^G93A^ mice increases the number of spinal cord microglia without affecting microglial activation status at disease end stage. **a** A flow cytometric analysis of wild-type (WT), SOD1^G93A^ (G93A), and SOD1^G93A^/TRIF^−/−^ (G93A/TKO) mouse spinal cord at disease end stage. CD45^low^/CD11b^+^ microglia were gated (orange frame). Representative forward scatter (FSC) versus side scatter (SSC) plots are shown. **b** Number of microglia in WT (*n* = 9), G93A (*n* = 7), and G93A/TKO (*n* = 6) spinal cord. **p < 0.01 (one-way ANOVA, Tukey–Kramer multiple comparison post hoc tests). Data are presented as mean ± SD. (**c**, **d**) Cellular size (FSC median: **c**) and granularity (SSC median: **d**) of CD45^low^/CD11b^+^ microglia compared to wild-type mice quantified by flow cytometric analysis from three mice per each genotype at disease end stage. Data are presented as mean ± SD. **e** Representative images of microglia stained for Iba1 (red) and DAPI (blue) in SOD1^G93A^ (G93A), SOD1^G93A^/TRIF^−/−^ (G93A/TKO), and SOD1^G93A^/MyD88^−/−^ (G93A/MKO) spinal cord at disease end stage. Scale bar, 100 μm. **f** An increased number of microglia was observed in SOD1^G93A^/TRIF^−/−^ mice (G93A/TKO, *n* = 7) compared to SOD1^G93A^ mice (G93A, *n* = 5) and SOD1^G93A^/MyD88^−/−^ mice (G93A/MKO, *n* = 3) at disease end stage. Iba1^+^/DAPI^+^ microglia were counted per unit area in the ventral horn of each spinal cord from section (six sections per mouse). Average number of microglia are plotted. Data are presented as mean ± SD. One-way ANOVA followed by Tukey–Kramer multiple comparison post hoc tests. **p < 0.01, ***p < 0.001. **g** Expression levels of activation markers (CD11c, CD68, CD86, CD206) and MHC (classes I and II) on CD45^low^/CD11b^+^ microglia were analyzed by flow cytometry at disease end stage. Representative histograms are shown. **h**–**m** Fold-changes in mean fluorescence intensity (MFI) compared to wild-type were quantified by flow cytometric analysis from at least three mice for each genotype. Data are presented as mean ± SD. Student’s *t *tests

To examine the influence of TRIF deficiency on the activation state of microglia, we performed flow cytometric and immunofluorescence analyses of spinal cord microglia among genotypes. Spinal cord microglia were identified by cell surface expression of CD45 and CD11b, and distinguished from lymphocytes and monocytes by lower levels of CD45 (Fig. 2a). Compared to wild-type, cell number, cell size, and granularity of CD45^low^/CD11b^+^ microglia were increased in SOD1^G93A^ mice (Fig. 2a–d). We also found an increase in the number of microglia in SOD1^G93A^ / TRIF^−/−^ mice compared to SOD1^G93A^ mice (Fig. 2b), although there was no difference in cellular size or granularity between the two genotypes (Fig. 2c, d). We then confirmed these results by immunofluorescence staining. The number of microglia was specifically increased in SOD1^G93A^/TRIF^−/−^ mice, but not in SOD1^G93A^/MyD88^−/−^ mice, compared to SOD1^G93A^ mice (Fig. 2e, f). However, we found that there were no differences in the expression levels of activation markers (CD11c, CD68), M1 (CD86) or M2 (CD206) microglial markers, or antigen-presenting cell maturation markers (major histocompatibility complex (MHC) classes I and II) between SOD1^G93A^ and SOD1^G93A^/TRIF^−/−^ spinal cord microglia at disease end stage (Fig. 2g–m). These data indicate that TRIF deficiency has little effects on the activation status of SOD1^G93A^ microglia.

### Decreased expression of chemokines CCL5 and CXCL10 in spinal cord microglia and astrocytes of SOD1^G93A^/TRIF^−/−^ mice

**Fig. 3:**
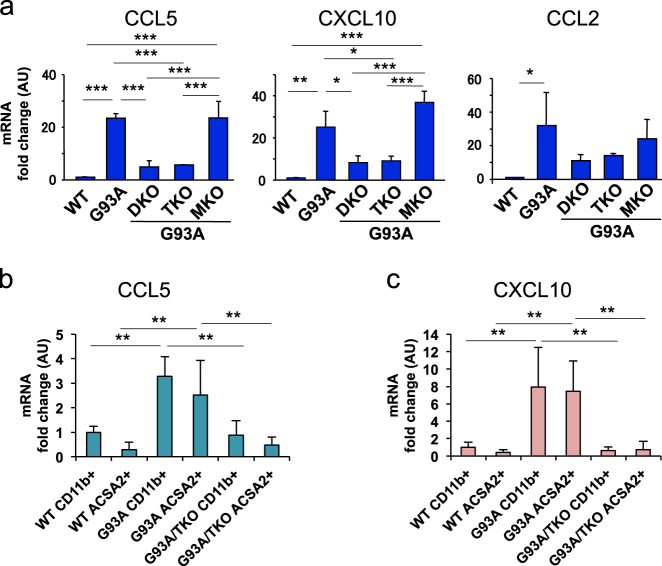
Decreased expression of the chemokines CCL5 and CXCL10 in TRIF-deficient SOD1^G93A^ spinal cord microglia and astrocytes at disease end stage. **a** mRNA levels of CCL5, CXCL10, and CCL2 in the lumbar spinal cord of end stage SOD1^G93A^ (G93A), SOD1^G93A^/TRIF^−/−^/MyD88^−/−^ (DKO), SOD1^G93A^/TRIF^−/−^ (TKO), and SOD1^G93A^/MyD88^−/−^ (MKO) mice as well as 5-month-old wild-type (WT) littermates determined by quantitative RT-PCR (*n* = 3 per genotypes). The mice were produced by a triple mating shown in Fig. 1e–h. Mean mRNA fold-changes relative to WT are plotted. Data are presented as mean ± SD. *p < 0.05, **p < 0.01, ***p < 0.001 (one-way ANOVA, Tukey–Kramer multiple comparison post hoc tests). **b**, **c** mRNA levels of CCL5 (**b**) and CXCL10 (**c**) in CD11b^+^ microglia and ACSA2^+^ astrocytes isolated from the spinal cord of end stage SOD1^G93A^ (G93A, *n* = 4 each) and SOD1^G93A^/TRIF^−/−^ (G93A/TKO, *n* = 4 each) mice as well as 5-month-old WT mice (WT, *n* = 5 each) determined by quantitative RT-PCR. Mean mRNA fold-changes relative to WT CD11b^+^ microglia are plotted. Data are presented as mean ± SD. **p < 0.01 (one-way ANOVA, Tukey–Kramer multiple comparison post hoc tests)

To identify dysregulated molecules in the spinal cord of TRIF-deficient SOD1^G93A^ mice, we quantified mRNA levels of cytokines, chemokines, neurotrophic factors, and other molecules implicated in ALS pathology. Among chemokines, mRNAs for CCL5 and CXCL10 were significantly upregulated in SOD1^G93A^ spinal cord. However, expression levels were markedly suppressed in SOD1^G93A^/TRIF^−/−^ and SOD1^G93A^/MyD88^−/−^/TRIF^−/−^ spinal cord (Fig. 3a). MyD88 deficiency alone did not change the expression levels of these chemokines in SOD1^G93A^ mice. Although mRNA levels for neurotrophic factors, cytokines, and chemokine receptors including IGF-I, GDNF, TGF-β, TNF-α, CD14, and CX3CR1 were upregulated in SOD1^G93A^ spinal cord, their expression levels were not affected when TRIF was eliminated (Figure S1). The mRNA levels of CCL2, IFN-β, and ISG15 were downregulated in SOD1^G93A^/TRIF^−/−^ mice (Fig. 3a, Figure S1), although the difference did not reach statistical significance. In summary, decreased expression levels of chemokines CCL5 and CXCL10 were specific to SOD1^G93A^/TRIF^−/−^ mice.

To identify the cell types responsible for TRIF-dependent production of these chemokines, we isolated microglia and astrocytes from the spinal cords of SOD1^G93A^ and SOD1^G93A^/TRIF^−/−^ mice by magnetic-activated cell sorting (MACS) and then performed quantitative reverse transcription polymerase chain reaction (RT-PCR). Expression levels of CCL5 and CXCL10 were elevated both in microglia and astrocytes of SOD1^G93A^ mice compared to wild-type mice, but significantly reduced in SOD1^G93A^/TRIF^−/−^ mice (Fig. 3b, c). Together, these data indicate that the TRIF pathway is activated in spinal cord microglia and astrocytes of SOD1^G93A^ mice, resulting in the elevated production of chemokines CCL5 and CXCL10.

### Decreased infiltration of T-lymphocytes and NK cells into the spinal cord of SOD1^G93A^/TRIF^−/−^ mice through reduced chemokine production

Previous reports suggested that a subpopulation of infiltrating T-lymphocytes into the spinal cord played a neuroprotective role in SOD1^G93A^ mice [14, 15]. Since TRIF-deficient microglia and astrocytes exhibited reduced production of chemokines CCL5 and CXCL10, we speculated that this may suppress the infiltration of immune cells into the spinal cord.

**Fig. 4:**
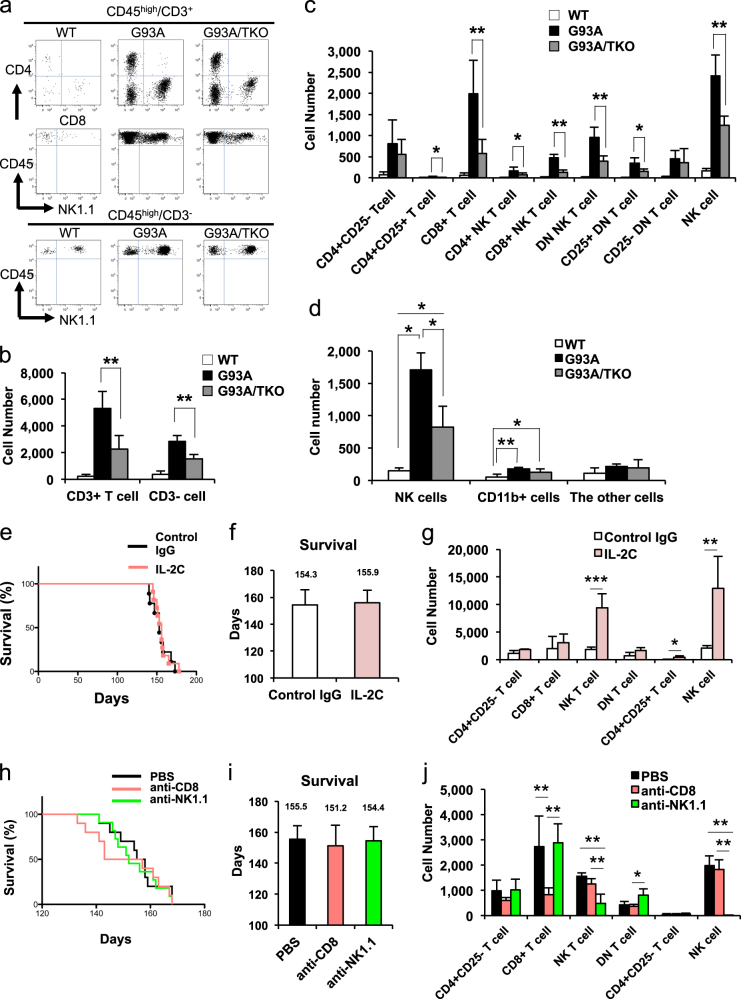
Manipulating the number of infiltrating CD8^+^-T, NK-T, and NK cells into spinal cord does not substantially alter the survival time of SOD1^G93A^ mice (**a**–**d**) Decreased infiltration of T-lymphocytes and natural killer (NK) cells into the spinal cords of SOD1^G93A^/ TRIF^−/−^ mice. **a** A flow cytometric analysis of wild-type (WT), SOD1^G93A^ (G93A), and SOD1^G93A^/TRIF^−/−^ (G93A/TKO) mouse spinal cord at disease end stage. CD45^high^/CD3^+^ cells and CD45^high^/CD3^−^ cells were gated. Representative plots for CD4, CD8, CD45, and NK1.1 are shown. **b**, **c** The number of infiltrated CD45^high^/CD3^+^ cells, CD45^high^/CD3^−^ cells, and subsets of immune cells isolated from spinal cord were quantified by flow cytometric analysis at disease end stage (WT: *n* = 10, SOD1^G93A^: *n* = 8, SOD1^G93A^/TRIF^−/−^: *n* = 9). Infiltrated CD45^high^/CD3^+^ cells and CD45^high^/CD3^−^ cells were significantly reduced in SOD1^G93A^/TRIF^−/−^ mouse spinal cord. CD8^+^ T cells, NK-T cells, and CD4^−^/CD8^−^ double negative (DN)-T cells as well as NK cells (CD3^−^) were significantly reduced in SOD1^G93A^/TRIF^−/−^ mouse spinal cord. Data are presented as mean ± SD. Statistical comparisons by Steel–Dwass tests or one-way ANOVA followed by Tukey–Kramer multiple comparison post hoc tests. *p < 0.05, **p < 0.01. **d** The subpopulation of CD45^high^/CD3^−^ cells in the spinal cord is plotted for wild-type (WT; *n* = 6), SOD1^G93A^ (G93A; *n* = 4), and SOD1^G93A^/TRIF^−/−^ (G93A/TKO; *n* = 6) mice. *p < 0.05, **p < 0.01. Data are presented as mean ± SD. Steel–Dwass tests or one-way ANOVA followed by Tukey–Kramer multiple comparison post hoc tests. **e**–**j** Altering the number of infiltrating CD8^+^-T, NK-T, and (or) NK cells only marginally affects the survival time of SOD1^G93A^ mice. **e** Survival curves of IL-2C-injected SOD1^G93A^ (red, *n* = 11) and control IgG-injected SOD1^G93A^ mice (black, *n* = 9). **f** Mean survival time of each genotype is plotted. Data are presented as mean ± SD. **g** The number of each infiltrated immune cell subset isolated from spinal cord were quantified by flow cytometric analysis at disease end stage (Control IgG: *n* = 5, IL-2C: *n* = 3). Infiltrated NK-T cells and NK cells were significantly increased in IL-2C-injected SOD1^G93A^ mouse spinal cord. Data are presented as mean ± SD and compared by Student’s *t* tests. *p < 0.05, **p < 0.01, ***p < 0.001. **h** Survival curves of anti-CD8-injected SOD1^G93A^ (red, *n* = 10), anti-NK1.1-injected SOD1^G93A^ (green, *n* = 11), and PBS-injected SOD1^G93A^ mice (black, *n* = 10). **i** Mean survival time of each genotype is plotted. Data are presented as mean ± SD. **j** The number of each infiltrated immune cell subset isolated from spinal cord were quantified by flow cytometric analysis at disease end stage (PBS: *n* = 4, anti-CD8: *n* = 5, anti-NK1.1: *n* = 6). Infiltrated CD8^+^-T cells were significantly reduced in anti-CD8-injected SOD1^G93A^ mouse spinal cords, while both NK-T and NK cells were significantly reduced in anti-NK1.1-injected SOD1^G93A^ mouse spinal cord. Data are presented as mean ± SD and analyzed by one-way ANOVA followed by Tukey–Kramer multiple comparison post hoc tests. *p < 0.05, **p < 0.01

To identify infiltrated immune cells in the spinal cord, we performed flow cytometric analysis for cell surface antigens at the disease end stage. The number of infiltrated CD45^high^/CD3^+^ cells (T-lymphocytes) and CD45^high^/CD3^−^ cells (non-T-lymphocytes) were reduced in SOD1^G93A^/TRIF^−/−^ spinal cord compared to SOD1^G93A^ spinal cord (Fig. 4a, b). Moreover, the majority of infiltrated lymphocytes in SOD1^G93A^ spinal cord were CD8^+^ T-lymphocytes (CD8^+^-T), natural killer T-lymphocytes (NK-T), and NK cells, all of which were significantly decreased in SOD1^G93A^/TRIF^−/−^ spinal cord (Fig. 4a, c). Notably, the number of infiltrated CD4^+^ T-lymphocytes and CD4^+^/CD25^+^ regulatory T-lymphocytes, which have been shown to be neuroprotective in ALS models [16, 26], were much smaller than those of CD8^+^-T, NK-T, or NK cells (Fig. 4c). Although one study reported that Ly6C^+^ monocytes infiltrated into the spinal cord of SOD1^G93A^ mice [27], our analysis revealed few infiltrated CD45^high^/CD11b^+^ monocytes (Fig. 4d), a finding in accord with the report by Chiu et al. [28]. These results suggest that the major populations of infiltrating lymphocytes are CD8^+^-T, NK-T, and NK cells and that infiltration of these cells depends on TRIF signaling.

### Altering the number of infiltrating CD8^+^-T, NK-T, and NK cells has little effect on the survival time of SOD1^G93A^ mice

To clarify the effects of infiltrating CD8^+^-T, NK-T, and NK cells on disease progression, we performed survival analyses of SOD1^G93A^ mice in which the number of these immune cells were altered from disease onset (100 days). Intraperitoneal (i.p.) injection of the mixture of IL-2 and anti-IL-2 antibody, known as IL-2 complex (IL-2C), increased the number of infiltrating NK-T and NK cells in the spinal cord of SOD1^G93A^ mice (Fig. 4g), in accord with previous results [29]. In contrast, i.p. injections of anti-CD8 or anti-NK1.1 antibody eliminated the infiltrating CD8^+^-T or NK-T and NK cells into the spinal cords of SOD1^G93A^ mice, respectively (Fig. 4j), again as shown previously [30, 31]. Although survival tended to be shorter in mice injected with anti-CD8, altering the total number of infiltrating immune cells had no effect on survival time (Fig. 4e, f, h, i). These results indicate that the infiltrating CD8^+^-T, NK-T, and NK cells contribute only marginally to the disease course in ALS mice.

### Increased number of abnormal Mac2-positive astrocytes with accumulation of p62 and ubiquitin in symptomatic SOD1^G93A^/TRIF^−/−^ mice

Activation of astrocytes and microglia, vacuolar pathology, oligodendrocyte dysfunction, and motor axon degeneration are well-known pathologies observed during disease progression in SOD1^G93A^ mice. Examination of the lumbar fifth motor roots revealed that the number and morphology of remaining axons were similar in end stage SOD1^G93A^ and SOD1^G93A^/TRIF^−/−^ mice (Figure S2a, b). The degree of vacuolar pathology, which reflects damaged mitochondria, also did not differ between genotypes (Figure S2c, d). In addition, there was no difference in the number of oligodendrocytes between genotypes at disease end stage (Figure S2e, f).

**Fig. 5:**
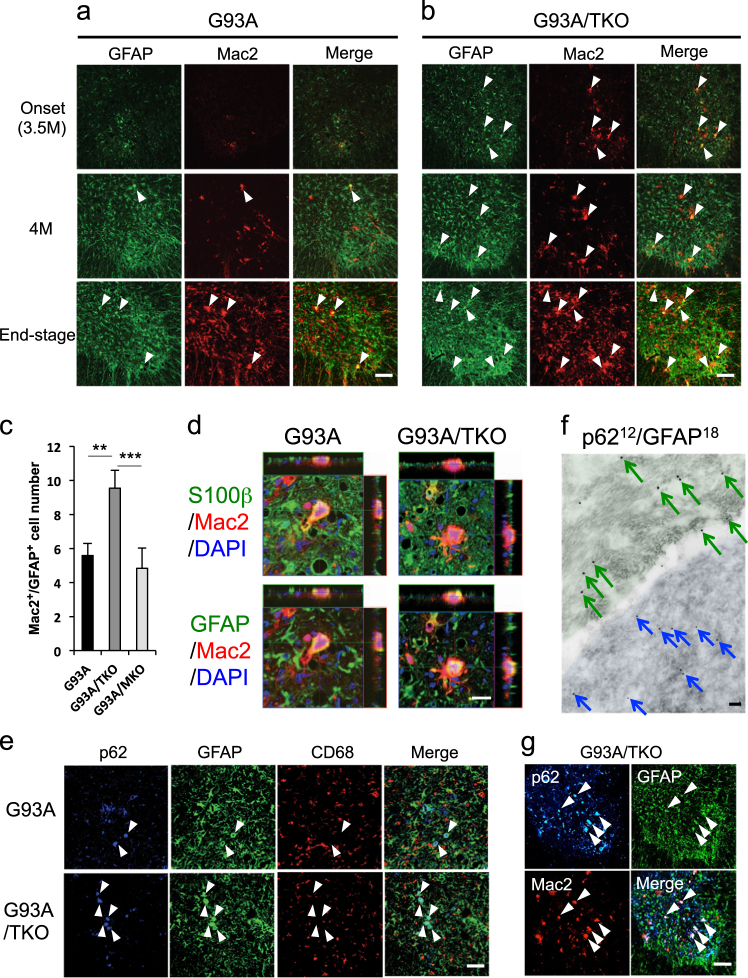
Accumulation of Mac2-expressing aberrant astrocytes in the lumbar spinal cord of symptomatic SOD1^G93A^/TRIF^−/−^ mice. **a**, **b** Activated microglia and astrocytes in the lumbar spinal cord of SOD1^G93A^ (G93A) (**a**) and SOD1^G93A^/TRIF^−/−^ (G93A/TKO) (**b**) mice at disease onset (3.5 months of age), 4 months of age, and disease end stage were visualized by immunostaining with antibodies for Mac2 and GFAP. Activated astrocytes expressing Mac2 (arrowheads) appeared earlier in SOD1^G93A^/TRIF^−/−^ mice than SOD1^G93A^ mice. Scale bar, 100 μm. **c** An increase in the number of Mac2-positive activated astrocytes was observed in SOD1^G93A^/TRIF^−/−^ mice (G93A/TKO) compared to SOD1^G93A^ mice (G93A) and SOD1^G93A^/MyD88^−/−^ mice (G93A/MKO) at disease end stage. Mac2^+^ astrocytes were counted per unit area of ventral horn in each spinal cord section (six sections per mouse) (G93A and G93A/TKO: *n* = 4 each, G93A/MKO: *n* = 3). Average number of Mac2^+^/GFAP^+^ astrocytes are plotted. Data are presented as mean ± SD and analyzed by one-way ANOVA followed by Tukey–Kramer multiple comparison post hoc tests. **p < 0.01, ***p < 0.001. **d** Representative three-dimensional images of astrocytes stained for S100β or GFAP (green), Mac2 (red), and DAPI (blue) in the lumbar spinal cord of SOD1^G93A^ (G93A) and SOD1^G93A^/TRIF^−/−^ (G93A/TKO) mice at disease end stage. Scale bar, 20 μm. **e** Images of the lumbar spinal cord of SOD1^G93A^ (G93A) and SOD1^G93A^/TRIF^−/−^ (G93A/TKO) mice at disease end stage stained for p62, GFAP, and CD68 along with the merged images. Arrowheads indicate the cells immunopositive for p62 and GFAP that do not express CD68. Scale bar, 50 μm. **f** A representative immune-electron microscopy image of the spinal cord astrocytes in SOD1^G93A^ mice at disease end stage. Large (green arrows) and small (blue arrows) gold particles label GFAP and p62, respectively. Scale bar, 500 nm. **g** Triple-staining of lumbar spinal cord of SOD1^G93A^/TRIF^−/−^ (G93A/TKO) mouse at early disease stage using antibodies for p62, GFAP, and Mac2. Arrowheads indicate p62-positive astrocytes expressing Mac2. Scale bar, 100 μm

On the other hand, double-immunofluorescence staining with GFAP and Mac2, markers of astrocytes and activated microglia, respectively, revealed greater number of Mac2-positive cells in the lumbar spinal cord of SOD1^G93A^/TRIF^−/−^ mice compared to SOD1^G93A^ mice after disease onset (Fig. 5a, b). More surprisingly, a substantial number of these Mac2-positive cells expressed GFAP, suggesting that these cells are in fact activated astrocytes (Fig. 5a–c). Moreover, accumulation of Mac2/GFAP-positive cells was not observed in SOD1^G93A^/MyD88^−/−^ mice (Fig. 5c). To confirm the identity of Mac2-positive cells, we also performed double-immunofluorescence staining with several astrocytic and microglial markers. Mac2-expressing cells with large and round cell bodies were immunopositive for the astrocytic markers GFAP and S100β (Fig. 5d) but negative for the microglial markers CD68 and Iba1 (Fig. 5e, Figure S3a–f). These results indicate that these Mac2-expressing cells are abnormally activated astrocytes similar to those previously described in mutant SOD1 rodents [32, 33].

Furthermore, we detected that intracellular accumulation of p62/sequestosome 1 and ubiquitin in most Mac2-expressing activated astrocytes by immunofluorescence and immunoelectron microscopy (Fig. 5e–g, Figure S3g–n). Elevated levels of p62 and ubiquitin reflect defects in autophagy-lysosome and ubiquitin-proteasomal degradation pathways, suggesting that these Mac2-positive astrocytes are functionally abnormal. Indeed, intracellular inclusions of human SOD1 were prominently observed in Mac2-expressing astrocytes (Figure S3o).

### TRIF pathway activation eliminates abnormal Mac2-positive astrocytes and remaining number negatively correlate with survival time of ALS mice

**Fig. 6:**
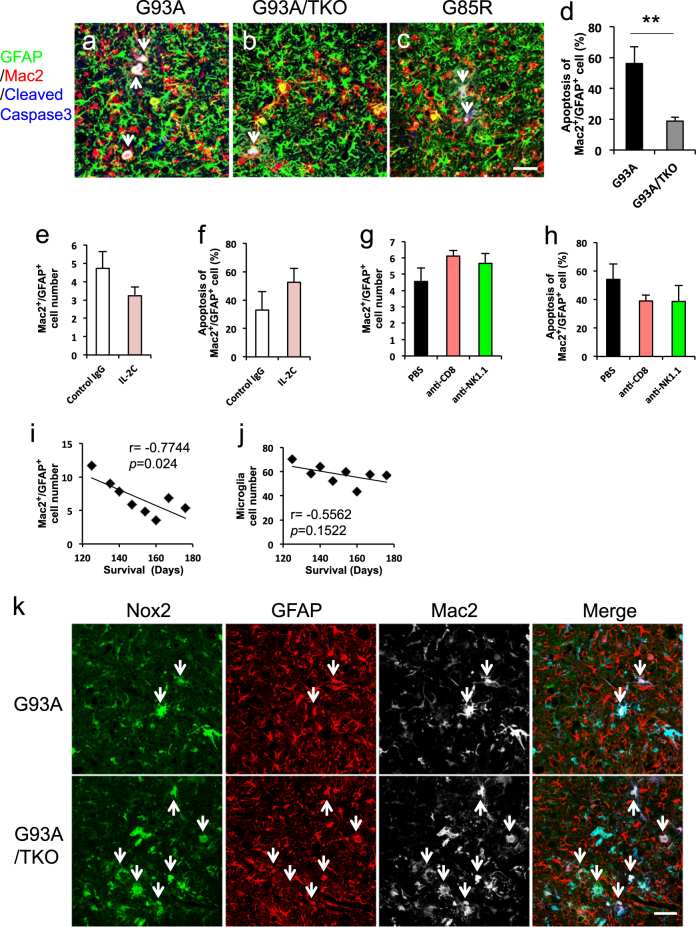
The TRIF pathway is involved in eliminating abnormal Mac2-positive astrocytes, and the number of these cells is negatively correlated with survival time of ALS mice. **a**–**c** Representative images of triple-immunofluorescence staining for GFAP (green), Mac2 (red), and cleaved caspase-3 (blue) in lumbar spinal cord sections from the indicated genotypes at disease end stage. Most Mac2-positive astrocytes were also immunopositive for cleaved caspase-3 (arrows) in SOD1^G93A^ (G93A) mice, but they were rarely immunopositive for cleaved caspase-3 (arrow) in SOD1^G93A^/TRIF^−/−^ (G93A/TKO) mice. Similar caspase-3-positive Mac2-expressing astrocytes were also observed in SOD1^G85R^ mice (G85R). Scale bar, 50 μm. **d** Percentages of cleaved caspase-3-positive Mac2-expressing astrocytes quantified by triple-immunofluorescence staining as shown in **a** and **b** (*n* = 4 per each genotype). Data are presented as mean ± SD and compared by Welch’s *t* tests. **p < 0.01. **e**, **g** The number of Mac2-positive astrocytes was not significantly altered in IL-2C-injected, anti-CD8-injected, or anti-NK1.1-injected SOD1^G93A^ mice as compared to control IgG-injected or PBS-injected mice at disease end stage. Average number of Mac2^+^/GFAP^+^ astrocytes is plotted. Data are presented as mean ± SD. **f**, **h** Percentages of cleaved caspase-3-positive Mac2-expressing astrocytes were quantified by triple-immunofluorescence staining for GFAP, Mac2, and cleaved caspase-3 (*n* = 3 per each genotype). Data presented as mean ± SD. Mac2^+^ astrocytes (**e**, **g**) or cleaved caspase-3^+^/Mac2^+^ astrocytes (**f**, **h**) were counted per unit area of ventral horn for each spinal cord section (six sections per mouse, *n* = 3 mice per each genotype). **i**, **j** Correlation plots (**i**) between average number of Mac2^+^/GFAP^+^ astrocytes and survival times (*r* = −0.7744 and *p* = 0.024) and (**j)** between average number of microglia and survival times (*r* = −0.5562 and *p* = 0.1522) in SOD1^G93A^/TRIF^−/−^ female mice (*n* = 8). Mac2^+^ astrocytes and microglia were counted per unit area of ventral horn for each spinal cord section (six sections per mouse) and correlations evaluated by Pearson’s correlation test. **k** Representative images of triple-immunofluorescence staining for Nox2 (green), GFAP (red), and Mac2 (white) in lumbar spinal cord sections from the indicated genotypes at disease end stage. Number of activated microglia (GFAP^−^/Mac2^+^ cells) and Mac2-positive astrocytes (GFAP^+^/Mac2^+^ cells, arrows) were increased in SOD1^G93A^/TRIF^−/−^ (G93A/TKO) mice and Mac2-positive astrocytes were also immunopositive for Nox2. Scale bar, 50 μm

Activation of the TLR-TRIF pathway but not the TLR-MyD88 pathway induces apoptosis in several cell types including microglia and macrophage [21, 34]. Thus, we examined apoptosis rates of activated microglia and Mac2-expressing astrocytes by triple-immunofluorescence staining for cleaved caspase-3, GFAP, and Mac2. Although we found very few apoptotic microglia, apoptosis was strongly induced in Mac2-expressing astrocytes of SOD1^G93A^ mouse spinal cord (Fig. 6a, arrows). However, the proportion of apoptotic Mac2-expressing astrocytes was significantly lower in SOD1^G93A^/TRIF^−/−^ mouse spinal cord compared to SOD1^G93A^ (Fig. 6b (arrows), d). Moreover, apoptotic Mac2-expressing astrocytes were also observed in another ALS model, the SOD1^G85R^ mouse (Fig. 6c). These results indicate that abnormal Mac2-expressing astrocytes are eliminated by apoptosis in SOD1 mutants with functional TRIF (SOD1^G93A^ and SOD1^G85R^), while elimination is incomplete in the absence of TRIF signaling (SOD1^G93A^/TRIF^−/−^ mice). The genotypic difference in astrocyte apoptosis rate appeared independent of the Fas cell death pathway induced by infiltrating cells. Expression of Fas was elevated in both Mac2-positive astrocytes (Figure S4a, arrows) and Mac2-negative astrocytes (Figure S4a, arrowheads) of SOD1^G93A^ and SOD1^G93A^/TRIF^−/−^ mouse spinal cord. In addition, infiltrating T-lymphocytes and NK cells but not microglia expressed the Fas receptor ligand FasL in both SOD1^G93A^ and SOD1^G93A^/TRIF^−/−^ spinal cord (Figure S4b, c). There were also no significant difference in the number and apoptosis rate of Mac2-expressing astrocytes among IL-2C-injected, anti-CD8-injected, or anti-NK1.1-injected SOD1^G93A^ mice (Fig. 6e–h), cohorts showing substantial differences in infiltrating cell populations (Fig. 4e–j). Collectively, these results indicate that infiltrating immune cells do not play a major role in eliminating Mac2-expressing astrocytes.

To clarify the relevance of Mac2-expressing astrocytes to disease progression, we analyzed the correlation between cell number and survival time of SOD1^G93A^/TRIF^−/−^ mice. The number of Mac2-expressing astrocytes in spinal cord was negatively correlated with survival time (Fig. 6i), whereas the number of microglia did not show any correlation with survival time (Fig. 6j). These results suggest that Mac2-expressing astrocytes rather than microglia contribute to disease progression.

Finally, to examine whether these activated astrocytes exacerbate neuroinflammation, we analyzed the expression of pro-inflammatory molecules by immunofluorescence staining. We found that NADPH-oxidase 2 (NOX2), known to be expressed in activated microglia and to generate reactive oxygen species (ROS), was strongly expressed in Mac2-expressing astrocytes in both SOD1^G93A^ and SOD1^G93A^/TRIF^−/−^ mice (Fig. 6k, arrows).

Taken together, these findings indicate that the TRIF pathway is involved in eliminating abnormally activated, neurotoxic Mac2-expressing astrocytes to control neuroinflammation, and maintain a microenvironment surrounding motor neurons.

## Discussion

In this study, we demonstrate that elimination of the TRIF-dependent TLR pathway accelerates disease progression of SOD1-ALS mice. At disease end stage, SOD1-ALS mice with TRIF deletion (SOD1^G93A^/TRIF^−/−^ mice) exhibited greater number of aberrantly activated astrocytes and microglia in the spinal cord compared to SOD1^G93A^ mice. Deficient TRIF signaling also reduced the number of infiltrating immune cells into the spinal cord, however, manipulation of infiltrating immune cells only marginally affected disease course. Alternatively, TRIF deficiency markedly reduced the apoptosis rate of these aberrantly activated astrocytes, and the number of these cells in spinal cord was negatively correlated with survival time. Collectively, our study indicates that the TRIF-dependent innate immune pathway has a novel beneficial role in controlling neuroinflammation by eliminating aberrantly activated astrocytes.

TLR signaling consists of two distinct pathways, TRIF-dependent and MyD88-dependent. A previous study reported that MyD88-deficient bone marrow transplantation accelerated disease progression in SOD1^G37R^ mice [23]. In contrast, by crossbreeding SOD1^G93A^ mice with MyD88- and TRIF-deficient mice we demonstrated that deficiency of TRIF, but not MyD88 deficiency, accelerates the disease progression in SOD1-ALS mice. Since complete elimination of MyD88 did not affect the survival time of SOD1^G37R^ mice [23], we speculate that the phenotype observed in chimeric SOD1^G37R^ mice with MyD88-deficient bone marrow is due to an off-target effect of the irradiation used to suppress hematopoiesis prior to transplantation [35]. Moreover, Optineurin and TANK-binding kinase 1 (TBK1), recently identified as causative genes for inherited ALS [36-38], are involved in TRIF/IRF3-mediated signaling [39, 40]. Based on these observations, we propose that TRIF-dependent modulation of neuroinflammation is a critical determinant of ALS progression.

Immune cells infiltrate the spinal cord in both human ALS patients and ALS model mice [14, 15, 41, 42]. Moreover, infiltrating T-lymphocytes conferred neuroprotection by modifying microglial activity in SOD1^G93A^ mice [14, 15]. However, altering the number of infiltrating CD8^+^-T, NK-T, and NK cells by administration of IL-2C or antibodies had only a marginally effect on the survival times of SOD1^G93A^ mice. Our results clearly indicate that the major populations of infiltrating immune cells had no substantial influence on disease course. Therefore, considering the results from previous studies and ours, the neuroprotective effects of T-lymphocytes may be mediated predominantly by CD4^+^-T cells.

We observed the accumulation of a peculiar subpopulation of astrocytes during disease progression co-expressing the microglial activation marker Mac2 and the astrocytic markers GFAP and S100β. Accumulation of p62, ubiquitin, and mutant SOD1 suggests that proteostasis is impaired in these astrocytes. These astrocytes possessed large round somata with fewer processes, and morphologically resembled the aberrant astrocytes previously reported to show degenerative phenotypes or neurotoxicity [32, 33]. These astrocytes also expressed cleaved caspase-3 and NOX2. The NADPH-oxidase NOX2 generates ROS, which are known to induce oxidative stress and one of the modifiers of ALS disease [43, 44]. Therefore, these abnormally activated astrocytes are likely to be harmful to spinal motor neurons by generating ROS. Indeed, correlation analysis revealed that greater number of Mac2-expressing astrocytes predicted shorter survival times of ALS mice. However, these cells were also undergoing concomitant apoptosis, the rate of which appeared to be determined by TRIF signaling. Considering that identification of similar subpopulation of Mac2-positive astrocytes has also been documented in cerebral ischemia models [45], our finding provide evidence of functionally and phenotypically distinct astrocytic subpopulation in ALS mice. The relevance of these astrocytes to other neurodegenerative diseases warrants further exploration.

Unlike MyD88-dependent TLR signaling, the TRIF-dependent TLR pathway induces apoptosis in multiple cell types including microglia and macrophages [21, 34]. In this study, we observed accumulation of aberrantly activated astrocytes and an increased number of activated microglia specifically in TRIF-deficient SOD1^G93A^ spinal cord. In addition, we also found that Mac2-expressing astrocytes robustly expressed NOX2 and that the number of such astrocytes was negatively correlated with the survival time of TRIF-deficient SOD1^G93A^ mice, whereas the number of microglia was not associated with survival. Therefore, we speculate that the activation of the TRIF pathway induces apoptosis of aberrantly activated astrocytes to mitigate oxidative stress and neuroinflammation surrounding motor neurons in the spinal cord. Indeed, apoptosis rate was significantly reduced in astrocytes of TRIF-deficient SOD1^G93A^ mice. We also examined the possible involvement of the Fas-FasL pathway in inducing apoptosis through infiltrating FasL-expressing immune cells. However, altering the number of infiltrating immune cells only marginally influenced the number of apoptotic astrocytes. It is still unclear how neuroinflammation mediated by glial activation is terminated. Our findings provide the first evidence that the TRIF pathway is involved in eliminating aberrantly activated astrocytes, thereby terminating the inflammatory response in the CNS. Elucidating of downstream signaling mechanisms controlling activated astrocytes apoptosis requires further study.

In conclusion, we reveal here that the TRIF-dependent innate immune pathway confers neuroprotection in ALS mice by eliminating aberrantly activated astrocytes. We expect that further investigation into the mechanisms controlling TRIF-dependent innate immunity in detrimental glial cells may provide novel candidate therapies for ALS.

## Materials and Methods

### Generation of mutant SOD1 mice deficient in MyD88 and (or) TRIF, and survival experiments

Transgenic mice expressing the familial ALS-linked SOD1^G93A^ gene (B6.Cg-Tg (SOD1*G93A)1Gur/J) on the C57BL/6 background were obtained from Jackson Laboratory (Bar Harbor, ME, USA) [46]. SOD1^G85R^ mice were kind gift from Dr. Cleveland (University of California, San Diego, CA, USA) [47]. MyD88^−/−^ and TRIF^−/−^ mice (on the C57BL/6 background) were generated as described previously [48, 49]. For double mating experiments (Figs. 1a–d, 2, 3b, 4–6, S2–4), mice heterozygous for the human SOD1^G93A^ transgene (SOD1^G93A^) were crossbred with mice heterozygous for *TRIF* or *MyD88* gene. Then, TRIF-deficient SOD1^G93A^ (SOD1^G93A^/TRIF^−/−^) or MyD88-deficient SOD1^G93A^ mice (SOD1^G93A^/ MyD88^−/−^) were generated by intercrossing SOD1^G93A^/TRIF^+/−^ mice with TRIF^+/−^ mice or SOD1^G93A^/MyD88^+/−^ mice with MyD88^+/−^ mice, respectively. For triple mating experiments (Figs. 1e–h, 3a, S1), mice heterozygous for the human SOD1^G93A^ transgene (SOD1^G93A^) were crossbred with mice heterozygous for MyD88 and TRIF genes (MyD88^+/−^/TRIF^+/−^) to produce SOD1^G93A^/MyD88^+/−^/TRIF^+/−^ mice. Subsequently, SOD1^G93A^ mice doubly deficient for MyD88 and TRIF (SOD1^G93A^/MyD88^−/−^/TRIF^−/−^), MyD88-deficient SOD1^G93A^ mice (SOD1^G93A^/ MyD88^−/−^), TRIF-deficient SOD1^G93A^ mice (SOD1^G93A^/TRIF^−/−^), and SOD1^G93A^ littermates were produced by intercrossing SOD1^G93A^/MyD88^+/−^/TRIF^+/−^ mice with MyD88^+/−^/TRIF^+/−^ mice. All of these genotypes were appeared at the expected probability of 3.1 % (1/32). The presence of the SOD1^G93A^ or SOD1^G85R^ transgene, MyD88 or TRIF genes was determined by PCR methods. Detailed protocols are provided in the following section.

For survival experiments, SOD1^G93A^ mice were always compared to their littermates without MyD88 or TRIF genes. Time of disease onset was retrospectively determined as the time when mice reached peak body weight and end stage was determined as the time when the animal could not right itself within 20 s after being placed on its side due to severe paralysis, an endpoint frequently used for mutant SOD1-expressing mice and one that was consistent with the requirements of the Animal Care and Use Committee of RIKEN and Nagoya University. Disease progression was defined by the duration between the onset and disease end stage. Statistical analysis of survival time and disease duration in Fig. 1 were performed with a log-rank tests with Bonferroni corrections for multiple comparisons and one-way analysis of variance (ANOVA) followed by Tukey–Kramer multiple comparison post hoc tests, respectively. These statistical analyses were conducted by using GraphPad Prism (GraphPad Software, USA).

### Genotyping of mice

Mice positive for the SOD1^G93A^ or SOD1^G85R^ transgene were identified by PCR using the following primers. The mouse SOD1 gene fragment (850 bp) was amplified by the mSOD-A primer (GTTACATATAGGGGTTTACTTCATAATCTG) and h/mSOD-C primer (CAGCAGTCACATTGCCCARGTCTCCAACATG). The human SOD1 gene fragment (750 bp) was amplified by the hSOD-B primer (CCAAGATGCTTAACTCTTGTAATCAATGGC) and h/mSOD-C primer. Mice deficient for the TRIF or MyD88 gene were identified by PCR using the following primers. The wild-type TRIF allele (300 bp) was amplified by the 5-TRIF primer (AGACCCTATGAACAGCATGTGTCACAG) and the 3-TRIF primer (CAAGATTGGACTTCACCTGGGTCCTTA), the targeted TRIF allele (350 bp) was amplified by the 5-TRIF primer and Neo-TRIF primer (CTAAAGCGCATGCTCCAGACTGCCTTG), the wild-type MyD88 allele (650 bp) was amplified by the 5-MyD88 primer (GAATCAGTCGCTTCTGTTGGACACCTG) and 3-MyD88 primer (CCGGCAACTAGAACAGACAGACTATCG), and the targeted MyD88 allele (350 bp) was amplified by the Neo-MyD88 primer (ATCGCCTTCTATCGCCTTCTTGACGAG) and 3-MyD88 primer.

### Immunofluorescence of spinal cord sections

The lumbar spinal cord was fixed by perfusion with 4% paraformaldehyde in phosphate buffer, cryoprotected for 48 h in 30 % sucrose in phosphate-buffered saline (PBS), and frozen. Twenty-micron or twelve-micron cryosections were immunostained with combinations of antibodies against the following proteins: GFAP (1:500, Dako, Denmark or 1:500, Sigma, USA), Mac2 (1:500, Cederlane, Canada), Iba1 (1:500, Wako, Japan), Iba1 (1:250, Novus Biologicals, USA), CD68 (1:500, Abcam, UK), p62 (1:1000, MBL, Japan), ubiquitin (1:500, Dako, Denmark), S100β (1:500, Swant, Switzerland), Fas (1:50, Enzo Life Sciences, USA), APC (cc-1) (1:200, Merck Millipore, USA), Nox2 (1:250, BD Biosciences, USA), hSOD1 (1:500, MBL, Japan), or cleaved caspase-3 (1:200, Cell Signaling, USA) overnight followed by fluorescently conjugated anti-rabbit, anti-rat, anti-mouse, or anti-goat immunoglobulin Gs (IgGs) (Thermo Fisher Scientific, USA or Jackson ImmunoResarch Laboratories, USA). For some of the antibodies, the tissue was pre-incubated with 5% normal goat serum or normal donkey serum/0.3% Triton X-100 in PBS for 1 h at room temperature prior to the incubation with primary antibodies. Sections were mounted with ProLong Antifade reagent (Thermo Fisher Scientific, USA) or VECTASHIELD mounting medium (VECTOR Laboratories, USA) and analyzed using a confocal microscope (LSM5 Exciter and LSM700, Carl-Zeiss, Germany).

### MACS of spinal cord microglia and astrocytes

The spinal cord, dissected from mice transcardially perfused with PBS, was dissociated at 37 °C for 15 min using the Neural Tissue Dissociation Kit—Postnatal Neurons (Miltenyi Biotec, Germany) by the gentleMACS Dissociator (Miltenyi Biotec, Germany). For isolation of microglia and astrocytes, myelin debris was removed by using Myelin Removal Beads II (Miltenyi Biotec, Germany). Purified cells were incubated with anti-CD16/CD32 antibodies (eBioscience, USA) for blocking Fc receptors, and then incubated with anti-CD11b microBeads (Miltenyi Biotec, Germany) for isolating microglia. CD11b-positive microglia were obtained by magnetic cell sorting through an LS column (Miltenyi Biotec, Germany). For isolating astrocytes, astrocyte-containing flow-through cells were incubated with anti-ACSA2 MicroBeads (Miltenyi Biotec, Germany), and then subjected to magnetic cell sorting through the LS column.

### Quantification of mRNA levels by RT-PCR

Total RNA was extracted from spinal cord or MACS sorted cells using TRIzol (Thermo Fisher Scientific, USA) and the RNeasy Mini Kit (Qiagen, Germany) or RNeasy Micro Kit (Qiagen, Germany) according to the manufacturer’s instructions. Complementary DNA (cDNA) from a spinal cord was generated from 1 µg of total RNA by using the PrimeScript II 1st strand cDNA Synthesis Kit (Takara Bio, Japan) and 1/20 of the yield was amplified using the Power SYBR Green PCR Master Mix (Applied Biosystems) in Thermal Cycler 7900HT (Thermo Fisher Scientific, USA). The thermocycle protocol was as follows: 1 cycle at 50 °C for 2 min, 1 cycle at 95 °C for 10 min, 50 cycles at 95 °C for 15 s and 60 °C for 1 min, and a dissociation stage of 95 °C for 15 s, 60 °C for 15 s, and 95 °C for 15 s. cDNA from MACS sorted cells was generated and amplified from 2.5 or 5 ng of total RNA by using the CellAmp Whole Transcriptome Amplification Kit (Real Time) Ver.2 (TaKaRa Bio, Japan) and 1/25 or 1/50 of the yield was amplified with the SYBR Premix Ex Taq II (Tli RNaseH Plus) (TaKaRa Bio, Japan) using the Thermal Cycler Dice Real Time System II or III (TaKaRa Bio, Japan). The thermocycle protocol was as follows: 1 cycle at 95 °C for 30 s, 40 cycles at 95 °C for 5 s and 60 °C for 30 s, and a dissociation stage of 95 °C for 15 s, 60 °C for 30 s, and 95 °C for 15 s. Mouse *beta-actin* or *glyceraldehyde-3-phosphate dehydrogenase* (*GAPDH*) was used for normalization. Every gene was run in duplicate together with the *beta-actin* or *GAPDH* control. Specific primers are listed in Supplementary Table 1. Statistical analysis of fold-changes in relative mRNA levels determined by real-time PCR was performed with one-way ANOVA followed by Tukey–Kramer multiple comparison post hoc tests using GraphPad Prism (GraphPad Software, USA).

### Flow cytometry analysis of spinal cord immune cells

The spinal cord, dissected from mice transcardially perfused with PBS, was minced into 1 mm^3^ pieces in solution containing 1 mg/mL or 0.1 mg/mL collagenase IV (Worthington Biochemical Corporation, USA) plus 0.4 mg/mL DNase I (Roche, Switzerland), and incubated at 37 °C for 15 min. For isolation of immune cells and microglia, cells were resuspended in 37% Percoll (GE Healthcare, USA) and centrifuged at 780 × *g* for 20 min. After centrifugation, myelin debris was removed and the cell pellet was collected. Cells were incubated with anti-CD16/CD32 antibodies (eBioscience, USA) for blocking Fc receptors, and then stained with combinations of the following antibodies: anti-CD45-PE-Cy7, anti-CD45-APC-Cy7, anti-CD4-PE, anti-CD8a-FITC, anti-CD8a-APC-Cy7, anti-NK1.1-PerCP-Cy5.5, anti-NK1.1-PE, anti-CD11c-PE, anti-CD11b-APC-Cy7, anti-CD11b-PerCP-Cy5.5 (BD Biosciences, USA), anti-CD4-APC, anti-CD3e-PerCP-Cy5.5, anti-CD86-APC, anti-CD25-PE (eBioscience, USA), anti-CD11c-APC, anti-I-A/I-E (MHC class II)-FITC, anti-CD4-PE-Cy7, anti-CD4-APC, anti-CD68-PE, anti-H-2K^b^/H-2D^b^ (MHC class I)-PE, anti-CD206-PE, anti-CD178 (FasL)-PE, and Armenian Hamster IgG Isotype control-PE (BioLegend, USA). Flow cytometry was performed by using FACS Aria and FACS Verse flow cytometers (BD Biosciences, USA) and the data was further analyzed using FlowJo Software (FlowJo, LLC, USA).

### Administration of cytokines and antibodies in vivo

The monoclonal antibodies against mouse IL-2, CD8, and NK1.1 were obtained from hybridoma clones, S4B6-1, 53-6.72, and PK-136, respectively (American Type Culture Collection, USA). The recombinant mouse IL-2 (rIL-2) was purchased from BioLegend (USA). For the preparation of IL-2C, 1.5 μg of rIL-2 and 15 μg of anti-IL-2 were mixed in vitro, and then IL-2C was i.p. injected two times per every week at 3-day or 4-day intervals from 100 days of age until endpoint. For the isotype control, the same amount of purified rat IgG2a (BioLegend) was used. For depletion of CD8^+^-T cells or NK-T cells and NK cells, 100 μg of anti-CD8 or anti-NK1.1 was i.p. injected two times per every week at 3-day or 4-day intervals from 100 days of age until endpoint. Equal volume of PBS was used as the control. The efficacies of these administrations were confirmed by flow cytometry.

### Quantification of microglia, astrocytes, and oligodendrocytes

To examine the cell number, the cellular size, the granularity, and the expression levels of activation markers of microglia by flow cytometry, CD45^low^/CD11b^+^ microglia were gated, and then analyzed by using FlowJo software. The cellular size and granularity were quantified as medians of forward scatter and side scatter histograms, respectively. The expression levels of activation markers were quantified as mean fluorescence intensities. All data are represented as fold-change compared to the average of wild-type mice. Fold-changes were compared by unpaired *t* test. Quantification of astrocyte, microglial, and oligodendrocyte number in serial sections of lumbar spinal cord was performed as follows. To determine the number of Mac2-expressing astrocyte, cells double-positive for Mac2 and GFAP were considered as Mac2-expressing astrocytes. To examine the apoptosis of Mac2-expressing astrocytes, we compared the ratios of triple-positive cells for Mac2, GFAP, and cleaved caspase-3 to double-positive cells for Mac2 and GFAP. Microglia were identified by Iba1 and DAPI staining and oligodendrocytes by APC (cc-1) and DAPI staining. The number of each cell type was counted per the unit area (320 μm × 320 μm) of ventral horn from every tenth 12 µm section across L4 to L5 spinal cord (about 120 µm distance between each section), corresponding to a total of six sections per mouse. Statistical analyses were performed by unpaired *t* tests or one-way ANOVA, Tukey–Kramer multiple comparison post hoc tests.

### Immunoelectron microscopy

Immunoelectron microscopy was performed on ultrathin cryosections as described previously [50]. Briefly, mice were deeply anesthetized with pentobarbital (25 mg/kg i.p.) and fixed by cardiac perfusion with 4% paraformaldehyde in 0.1 M phosphate buffer (PB) (pH 7.2). Spinal cords (mid lumbar segment) were quickly removed, and transverse sections of about 1 mm in thickness were cut and immersed in the same fixative overnight at 4 °C. After being washed thoroughly with 7.5% sucrose in 0.1 M PB (pH 7.2), the samples were embedded in 12% gelatin, rotated in 2.3 M sucrose in 0.1 M PB overnight at 4 °C, placed on a specimen holder (Leica, Germany), and quickly plunged into liquid nitrogen until sectioning. Ultrathin cryosections were cut with a Leica UC7/FC7 at about –120 °C. Sections of about 70 nm thickness were picked up with a 1:1 mixture of 2% methylcellulose and 2.3 M sucrose and transferred to a nickel grid bearing a carbon-coated Formvar supporting film. The sections were rinsed with PBS containing 0.02 M glycine, treated with 1% bovine serum albumin in PBS, and incubated overnight at 4 °C with a mixture of rabbit anti-p62 (1:20, Wako, Japan) and goat anti-GFAP (1:20, Frontier Science, Japan), following the incubation with a mixture of donkey anti-rabbit and goat IgG conjugated with 12 and 18 nm colloidal gold particles, respectively (1:30 each, Jackson ImmunoResarch Laboratories, West Grove, PA, USA) for 1 h at room temperature. After labeling, the sections were embedded in a thin layer of 2% methylcellulose with 0.4 % uranyl acetate (pH 4.0), air-dried, and then observed with a Hitachi H-7100 electron microscope (Hitachi, Japan). For the control experiments, ultrathin sections were reacted only with the gold particle-conjugated secondary antibody.

### Semi-thin sections of spinal cord and roots

Spinal cords, which were fixed by perfusion with 2% glutaraldehyde–2% paraformaldehyde buffered with 0.1 M PB (pH 7.2) and further immersed in the same fixatives for 2 h, were transversely sectioned into 1 mm blocks. Lumbar roots, treated in the same methods as spinal cords, were not sectioned before postfixation with 2% osmium tetroxide. Samples were postfixed with 2% osmium tetroxide buffered with 0.1 M PB (pH 7.2), dehydrated with a graded series of alcohol, and embedded in Epon 812 (Taab Laboratories Equipment, UK). One-micrometer sections were cut with an ultramicrotome Leica EM UC6 (Leica, Germany) and stained with toluidine blue.

### Statistical analysis

Survival time was analyzed by log-rank tests with Bonferroni correction for multiple comparisons and disease duration by one-way ANOVA followed by Tukey–Kramer multiple comparison post hoc tests. Correlations were assessed by Pearson’s correlation test. All other data were analyzed by unpaired *t* test, one-way ANOVA, Tukey–Kramer multiple comparison post hoc tests, Welch’s *t* test, or Steel–Dwass tests as indicated. The analyses were performed using GraphPad Prism (GraphPad Software, La Jolla, CA, USA) or add-in software Statcel 3 (OMS, Tokyo, Japan). Data are presented as mean ± SD (standard deviation). The number of biological replicates is presented as “*n*” in the text. *P* values of <0.05 were considered statistically significant for all tests.

## Electronic supplementary material

non-highlighted Supplemetary text(DOCX 92 kb)

Figure S1, S2, S3, and S4, Table S1(PDF 6060 kb)

## References

[1] Lucin KM, Wyss-Coray T (2009). Immune activation in brain aging and neurodegeneration: too much or too little?. Neuron.

[2] Prinz M, Priller J, Sisodia SS, Ransohoff RM (2011). Heterogeneity of CNS myeloid cells and their roles in neurodegeneration. Nat Neurosci.

[3] Ransohoff RM, Brown MA (2012). Innate immunity in the central nervous system. J Clin Invest.

[4] Heneka MT, Carson MJ, El Khoury J, Landreth GE, Brosseron F, Feinstein DL (2015). Neuroinflammation in Alzheimer’s disease. Lancet Neurol.

[5] Ilieva H, Polymenidou M, Cleveland DW (2009). Non-cell autonomous toxicity in neurodegenerative disorders: ALS and beyond. J Cell Biol.

[6] Komine O, Yamanaka K (2015). Neuroinflammation in motor neuron disease. Nagoya J Med Sci.

[7] Yamanaka K, Komine O (2018). The multi-dimensional roles of astrocytes in ALS. Neurosci Res.

[8] Boillee S, Yamanaka K, Lobsiger CS, Copeland NG, Jenkins NA, Kassiotis G (2006). Onset and progression in inherited ALS determined by motor neurons and microglia. Science.

[9] Beers DR, Henkel JS, Xiao Q, Zhao W, Wang J, Yen AA (2006). Wild-type microglia extend survival in PU.1 knockout mice with familial amyotrophic lateral sclerosis. Proc Natl Acad Sci USA.

[10] Wang L, Sharma K, Grisotti G, Roos RP (2009). The effect of mutant SOD1 dismutase activity on non-cell autonomous degeneration in familial amyotrophic lateral sclerosis. Neurobiol Dis.

[11] Yamanaka K, Chun SJ, Boillee S, Fujimori-Tonou N, Yamashita H, Gutmann DH (2008). Astrocytes as determinants of disease progression in inherited amyotrophic lateral sclerosis. Nat Neurosci.

[12] Wang L, Gutmann DH, Roos RP (2011). Astrocyte loss of mutant SOD1 delays ALS disease onset and progression in G85R transgenic mice. Hum Mol Genet.

[13] Kang SH, Li Y, Fukaya M, Lorenzini I, Cleveland DW, Ostrow LW (2013). Degeneration and impaired regeneration of gray matter oligodendrocytes in amyotrophic lateral sclerosis. Nat Neurosci.

[14] Beers DR, Henkel JS, Zhao W, Wang J, Appel SH (2008). CD4+T cells support glial neuroprotection, slow disease progression, and modify glial morphology in an animal model of inherited ALS. Proc Natl Acad Sci USA.

[15] Chiu IM, Chen A, Zheng Y, Kosaras B, Tsiftsoglou SA, Vartanian TK (2008). T lymphocytes potentiate endogenous neuroprotective inflammation in a mouse model of ALS. Proc Natl Acad Sci USA.

[16] Beers DR, Henkel JS, Zhao W, Wang J, Huang A, Wen S (2011). Endogenous regulatory T lymphocytes ameliorate amyotrophic lateral sclerosis in mice and correlate with disease progression in patients with amyotrophic lateral sclerosis. Brain: a J Neurol.

[17] Banerjee R, Mosley RL, Reynolds AD, Dhar A, Jackson-Lewis V, Gordon PH (2008). Adaptive immune neuroprotection in G93A-SOD1 amyotrophic lateral sclerosis mice. PLoS ONE.

[18] Carpentier PA, Duncan DS, Miller SD (2008). Glial toll-like receptor signaling in central nervous system infection and autoimmunity. Brain Behav Immun.

[19] Kielian T (2006). Toll-like receptors in central nervous system glial inflammation and homeostasis. J Neurosci Res.

[20] Kawai T, Akira S (2010). The role of pattern-recognition receptors in innate immunity: update on Toll-like receptors. Nat Immunol.

[21] Ruckdeschel K, Pfaffinger G, Haase R, Sing A, Weighardt H, Hacker G (2004). Signaling of apoptosis through TLRs critically involves toll/IL-1 receptor domain-containing adapter inducing IFN-beta, but not MyD88, in bacteria-infected murine macrophages. J Immunol.

[22] Lehnardt S (2010). Innate immunity and neuroinflammation in the CNS: the role of microglia in Toll-like receptor-mediated neuronal injury. Glia.

[23] Kang J, Rivest S (2007). MyD88-deficient bone marrow cells accelerate onset and reduce survival in a mouse model of amyotrophic lateral sclerosis. J Cell Biol.

[24] Zhao W, Beers DR, Henkel JS, Zhang W, Urushitani M, Julien JP (2010). Extracellular mutant SOD1 induces microglial-mediated motoneuron injury. Glia.

[25] Lee JY, Lee JD, Phipps S, Noakes PG, Woodruff TM (2015). Absence of toll-like receptor 4 (TLR4) extends survival in the hSOD1 G93A mouse model of amyotrophic lateral sclerosis. J Neuroinflamm.

[26] Henkel JS, Beers DR, Wen S, Rivera AL, Toennis KM, Appel JE (2013). Regulatory T-lymphocytes mediate amyotrophic lateral sclerosis progression and survival. EMBO Mol Med.

[27] Butovsky O, Siddiqui S, Gabriely G, Lanser AJ, Dake B, Murugaiyan G (2012). Modulating inflammatory monocytes with a unique microRNA gene signature ameliorates murine ALS. J Clin Invest.

[28] Chiu IM, Morimoto ET, Goodarzi H, Liao JT, O’Keeffe S, Phatnani HP (2013). A neurodegeneration-specific gene-expression signature of acutely isolated microglia from an amyotrophic lateral sclerosis mouse model. Cell Rep.

[29] Boyman O, Kovar M, Rubinstein MP, Surh CD, Sprent J (2006). Selective stimulation of T cell subsets with antibody–cytokine immune complexes. Science.

[30] Cobbold SP, Jayasuriya A, Nash A, Prospero TD, Waldmann H (1984). Therapy with monoclonal antibodies by elimination of T-cell subsets in vivo. Nature.

[31] Koo GC, Dumont FJ, Tutt M, Hackett J, Kumar V (1986). The NK-1.1(−) mouse: a model to study differentiation of murine NK cells. J Immunol.

[32] Rossi D, Brambilla L, Valori CF, Roncoroni C, Crugnola A, Yokota T (2008). Focal degeneration of astrocytes in amyotrophic lateral sclerosis. Cell Death Differ.

[33] Diaz-Amarilla P, Olivera-Bravo S, Trias E, Cragnolini A, Martinez-Palma L, Cassina P (2011). Phenotypically aberrant astrocytes that promote motoneuron damage in a model of inherited amyotrophic lateral sclerosis. Proc Natl Acad Sci USA.

[34] Kim SJ, Li J (2013). Caspase blockade induces RIP3-mediated programmed necrosis in Toll-like receptor-activated microglia. Cell Death Dis.

[35] Ajami B, Bennett JL, Krieger C, Tetzlaff W, Rossi FM (2007). Local self-renewal can sustain CNS microglia maintenance and function throughout adult life. Nat Neurosci.

[36] Cirulli ET, Lasseigne BN, Petrovski S, Sapp PC, Dion PA, Leblond CS (2015). Exome sequencing in amyotrophic lateral sclerosis identifies risk genes and pathways. Science.

[37] Freischmidt A, Wieland T, Richter B, Ruf W, Schaeffer V, Muller K (2015). Haploinsufficiency of TBK1 causes familial ALS and fronto-temporal dementia. Nat Neurosci.

[38] Maruyama H, Morino H, Ito H, Izumi Y, Kato H, Watanabe Y (2010). Mutations of optineurin in amyotrophic lateral sclerosis. Nature.

[39] Sakaguchi T, Irie T, Kawabata R, Yoshida A, Maruyama H, Kawakami H (2011). Optineurin with amyotrophic lateral sclerosis-related mutations abrogates inhibition of interferon regulatory factor-3 activation. Neurosci Lett.

[40] Sato S, Sugiyama M, Yamamoto M, Watanabe Y, Kawai T, Takeda K (2003). Toll/IL-1 receptor domain-containing adaptor inducing IFN-beta (TRIF) associates with TNF receptor-associated factor 6 and TANK-binding kinase 1, and activates two distinct transcription factors, NF-kappa B and IFN-regulatory factor-3, in the Toll-like receptor signaling. J Immunol.

[41] Engelhardt JI, Tajti J, Appel SH (1993). Lymphocytic infiltrates in the spinal cord in amyotrophic lateral sclerosis. Arch Neurol.

[42] Troost D, van den Oord JJ, de Jong JM, Swaab DF (1989). Lymphocytic infiltration in the spinal cord of patients with amyotrophic lateral sclerosis. Clin Neuropathol.

[43] Marden JJ, Harraz MM, Williams AJ, Nelson K, Luo M, Paulson H (2007). Redox modifier genes in amyotrophic lateral sclerosis in mice. J Clin Invest.

[44] Marrali G, Casale F, Salamone P, Fuda G, Caorsi C, Amoroso A (2014). NADPH oxidase (NOX2) activity is a modifier of survival in ALS. J Neurol.

[45] Morizawa YM, Hirayama Y, Ohno N, Shibata S, Shigetomi E, Sui Y (2017). Reactive astrocytes function as phagocytes after brain ischemia via ABCA1-mediated pathway. Nat Commun.

[46] Gurney ME, Pu H, Chiu AY, Dal Canto MC, Polchow CY, Alexander DD (1994). Motor neuron degeneration in mice that express a human Cu,Zn superoxide dismutase mutation. Science.

[47] Bruijn LI, Becher MW, Lee MK, Anderson KL, Jenkins NA, Copeland NG (1997). ALS-linked SOD1 mutant G85R mediates damage to astrocytes and promotes rapidly progressive disease with SOD1-containing inclusions. Neuron.

[48] Yamamoto M, Sato S, Hemmi H, Hoshino K, Kaisho T, Sanjo H (2003). Role of adaptor TRIF in the MyD88-independent toll-like receptor signaling pathway. Science.

[49] Adachi O, Kawai T, Takeda K, Matsumoto M, Tsutsui H, Sakagami M (1998). Targeted disruption of the MyD88 gene results in loss of IL-1- and IL-18-mediated function. Immunity.

[50] Koike M, Shibata M, Sunabori T, Yamaguchi J, Sakimura K, Komatsu M (2017). Purkinje cells are more vulnerable to the specific depletion of cathepsin D than to that of Atg7. Am J Pathol.

