# Supplementary material for: Innate immune adaptor TRIF deficiency accelerates disease progression of ALS mice with accumulation of aberrantly activated astrocytes
Source: Cell Death Differ. 2018 Mar 22;25(12):2130–46. doi: 10.1038/s41418-018-0098-3 (PMC6261996; doi:10.1038/s41418-018-0098-3)
Supplement: Supplementary file 1 — non-highlighted Supplemetary text(DOCX 92 kb) [file 41418_2018_98_MOESM1_ESM.docx]

**Supplementary Information**

**Innate immune adaptor TRIF deficiency accelerates disease progression of ALS mice with accumulation of aberrantly activated astrocytes**

Okiru Komine, Hirofumi Yamashita, Noriko Fujimori-Tonou, Masato Koike, Shijie Jin, Yasuhiro Moriwaki, Fumito Endo, Seiji Watanabe, Satoshi Uematsu, Shizuo Akira, Yasuo Uchiyama, Ryosuke Takahashi, Hidemi Misawa, Koji Yamanaka

**Supplementary Figures**

Figure S1: Relative mRNA expression levels of chemokines, cytokines, neurotrophic factors, inflammation-related molecules in the lumbar spinal cord of SOD1^G93A^ mice with or without MyD88 or TRIF

Figure S2: Histological evaluation of motor neurons, axons, and oligodendrocytes in SOD1^G93A^ mice with or without TRIF

Figure S3: Mac2-positive cells accumulated in SOD1^G93A^/TRIF^−/−^ spinal cords show abnormal aggregations of p62, ubiquitin, and mutant hSOD1 without expression of a microglia marker Iba-1

Figure S4: Fas expression in abnormal Mac2-positive astrocytes and FasL expression in infiltrating immune cells were observed in both SOD1^G93A^ and SOD1^G93A^/TRIF^−/−^ spinal cords

**Supplementary Table**

Table S1: Oligonucleotides used in this study.

**Supplementary Figure Legends**

**Figure S1.** **Relative mRNA expression levels of chemokines, cytokines, neurotrophic factors, inflammation-related molecules in the lumbar spinal cord of SOD1^G93A^ mice with or without MyD88 or TRIF**

(a) Relative mRNA levels of inflammation-related molecules determined by quantitative RT-PCR. WT: non-transgenic mice, G93A: SOD1^G93A^ mice, DKO: SOD1^G93A^/MyD88^−/−^/TRIF^−/−^ mice, TKO: SOD1^G93A^/TRIF^−/−^ mice, MKO: SOD1^G93A^/MyD88^−/−^ mice. Data presented as mean ± SD and analyzed by one-way ANOVA followed by Tukey-Kramer multiple comparison post hoc tests. n.s.: not significant. *p < 0.05. (b) Summary of mRNA expression levels (SOD1^G93A^/TRIF^−/−^ vs. SOD1^G93A^) of Figure 3 a and Figure S1 a. ↓↓: >50 % decrease, N.S.: not significant. *: p < 0.05.

**Figure S2. Histological evaluation of motor neurons, axons, and oligodendrocytes in SOD1^G93A^ mice with or without TRIF**

(a) Representative micrograph of lumbar 5^th^ ventral roots (stained with Toluidine blue) from end stage SOD1^G93A^ (G93A) and SOD1^G93A^/TRIF^−/−^ (G93A/TKO) mice. (b) Average numbers of lumbar 5^th^ motor axons from end stage SOD1^G93A^ (G93A) and SOD1^G93A^/TRIF^−/−^ (G93A/TKO) mice along with 5 month old TRIF^−/−^ (TKO) mice (n=3, each) are plotted. Data presented as mean ± SD and analyzed by one-way ANOVA followed by Tukey-Kramer multiple comparison post hoc tests. ***p < 0.001. n.s.: not significant. (c, d) Semi-thin section of the ventral lumbar spinal cords of symptomatic SOD1^G93A^ (G93A) (c) and SOD1^G93A^/TRIF^−/−^ (G93A/TKO) (d) mice were stained with Toluidine blue. The degree of vacuolar pathology, which reflects damaged mitochondria, was unchanged between genotypes. Scale bar, 25 μm. **(**e**)** Representative images of oligodendrocytes stained for APC (cc-1) (red) in the lumbar spinal cords of SOD1^G93A^ (G93A) and SOD1^G93A^/TRIF^−/−^ (G93A/TKO) mice at disease end stage and of a 5-month-old wild-type (WT) mouse. Scale bar, 100 μm. (f) There were no differences in the number of oligodendrocytes among genotypes. APC (cc-1)^+^ oligodendrocytes were counted per unit area of ventral horn in each spinal cord section (6 sections per mouse) (n = 3 each). Average numbers of oligodendrocytes are plotted. Data presented as mean ± SD.

**Figure S3. Mac2-positive cells accumulated in SOD1^G93A^/TRIF**^−/−^ **spinal cords show abnormal aggregations of p62, ubiquitin, and mutant hSOD1 without expression of a microglia marker Iba-1**

(a-f) Mac2-positive cells do not express microglia marker, Iba-1. Representative images of double-immunofluorescence staining for Iba-1 (green: a, d) and Mac2 (red: b, e) along with the merged images (c, f) in the lumbar spinal cord of SOD1^G93A^ (G93A: a-c) and SOD1^G93A^/TRIF^−/−^ (G93A/TKO: d-f) mice. Mac2^+^/Iba-1^-^ cells (arrows), which were especially increased in SOD1^G93A^/TRIF^−/−^ mice were observed in both genotypes. Bars: 50 μm. (g-n) Mac2-positive abnormal cells are immunopositive for ubiquitin and p62. The images of lumbar spinal cord of SOD1^G93A^ (G93A) (g-j) and SOD1^G93A^/TRIF^−/−^ (G93A/TKO) (k-n) mice at early disease stage using antibodies for ubiquitin (green: g, k), p62 (red: h, l), and Mac2 (blue: i, m). Merged images (j, n) have DAPI (pink) stainings as well as the stainings using three antibodies. Arrows indicate Ubiquitin/p62/Mac2 triple-positive cells. p62-positive inclusion in Mac2-expressing cells is also immunopositive for ubiquitin. Scale bars, 50 μm. (o) The aggregation of mutant human SOD1 also observed in Mac2-positive abnormal cells which expressed an astrocyte marker GFAP. Scale bar, 100 μm.

**Figure S4. Fas expression in abnormal Mac2-positive astrocytes and FasL expression in infiltrating immune cells were observed in both SOD1^G93A^ and SOD1^G93A^/TRIF**^−/−^ **spinal cords**

(a) Triple-immunofluorescence staining for Fas (green), GFAP (red), and Mac2 (white) in lumbar spinal cord sections of SOD1^G93A^ (G93A) and SOD1^G93A^/TRIF^−/−^ (G93A/TKO) mice at disease end stage and of a 5-month-old wild-type (WT) mouse. Increased Fas expression was observed in both Mac2-positive (arrows) and Mac2-negative (arrowheads) astrocytes. Scale bar, 50 μm. (b) A flow cytometric analysis of FasL expressions of infiltrating immune cells and microglia isolated from spinal cords of SOD1^G93A^ (G93A) and SOD1^G93A^/TRIF^−/−^ (G93A/TKO) mice at disease end stage. The red lines represent fluorescence intensity of FasL expression, whereas the gray lines represent fluorescence intensity of control IgG (isotype control). Percentages of FasL positive cells per total cells were shown as number. (c) Percentages of FasL-positive cells in infiltrating immune cells and microglia were quantified by a flow cytometric analysis (SOD1^G93A^ (G93A): n = 5, SOD1^G93A^/TRIF^−/−^ (G93A/TKO): n = 4).

**Table S1. Oligonucleotides used in this study**
