# Supplementary material for: Innate immune adaptor TRIF deficiency accelerates disease progression of ALS mice with accumulation of aberrantly activated astrocytes
Source: Cell Death Differ. 2018 Mar 22;25(12):2130–46. doi: 10.1038/s41418-018-0098-3 (PMC6261996; doi:10.1038/s41418-018-0098-3)
Supplement: Supplementary file 2 — Figure S1, S2, S3, and S4, Table S1(PDF 6060 kb) [file 41418_2018_98_MOESM2_ESM.pdf]

Figure S1

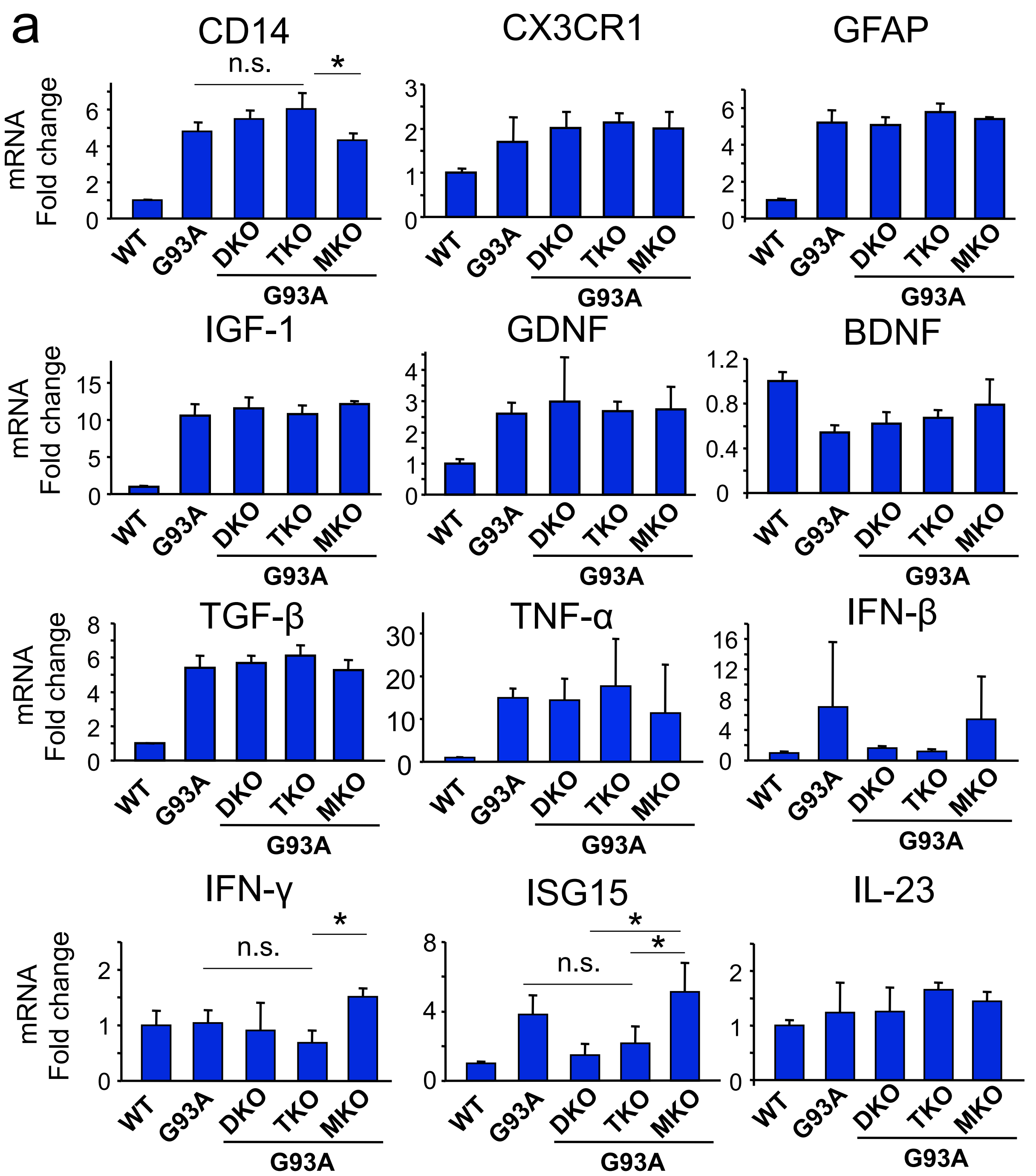

b

mRNA levels of SOD1^{G93A}/TRIF$^{-/-}$ vs SOD1^{G93A}

1. Chemokines and receptors
- CCL5: ↓↓ *
- CXCL10: ↓↓ *
- CCL2: N.S.
- CX3CR1: N.S.

2. Neurotrophic factors
- IGF-1: N.S.
- BDNF: N.S.
- GDNF: N.S.

3. Cytokines, inflammation
- TNF-α: N.S.
- TGF-β1: N.S.
- IFN-β: N.S.
- IFN-γ: N.S.
- ISG15: N.S.
- IL-23: N.S.

* : $P < 0.05$

Figure S2

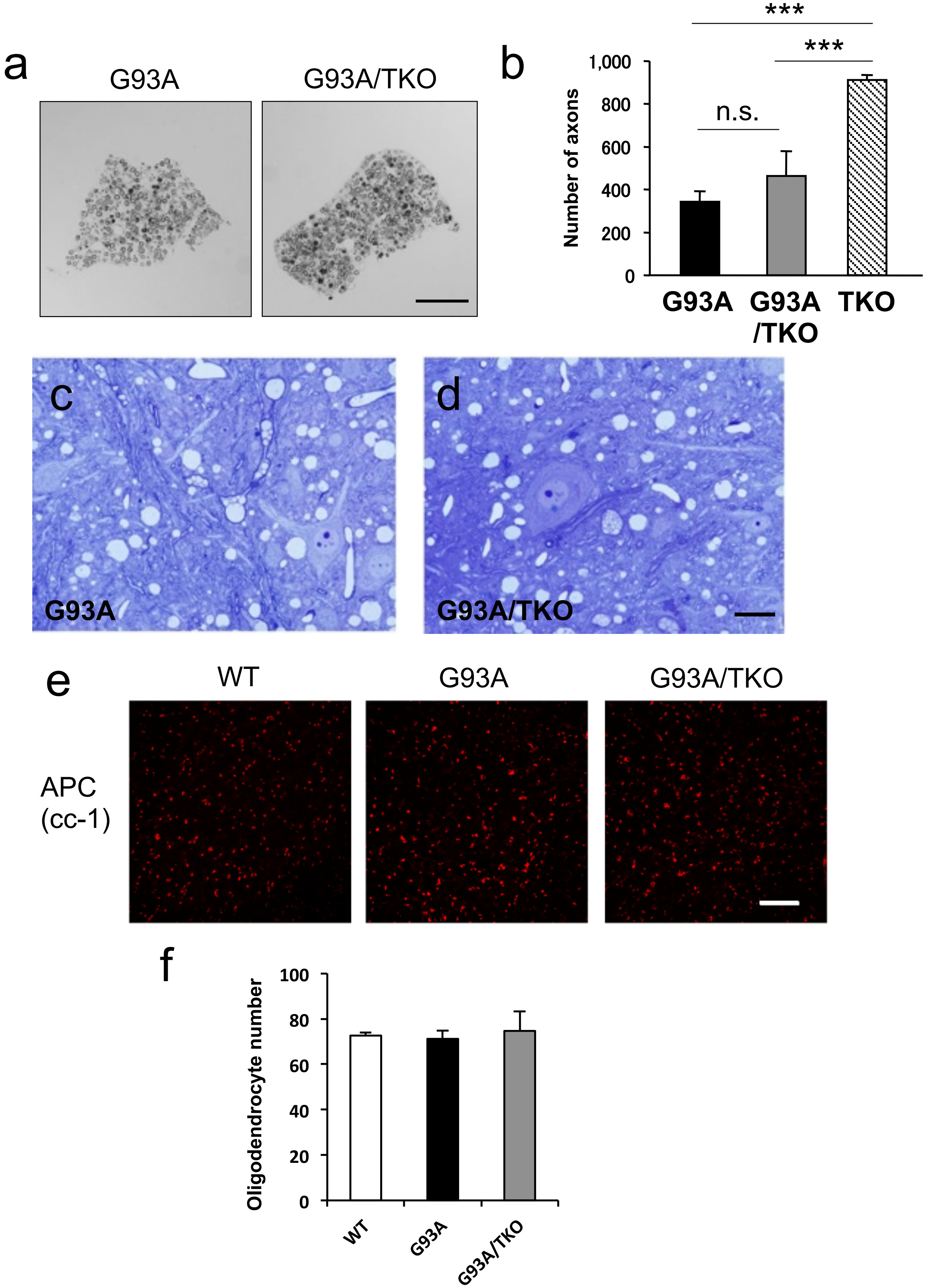
a
G93A
G93A/TKO
b
***
***
1,000
800
600
400
200
0
n.s.
Number of axons
G93A
G93A /TKO
TKO
c
G93A
d
G93A/TKO
e
WT
G93A
G93A/TKO
APC (cc-1)
f
100
80
60
40
20
0
Oligodendrocyte number
WT
G93A
G93A/TKO

Figure S3

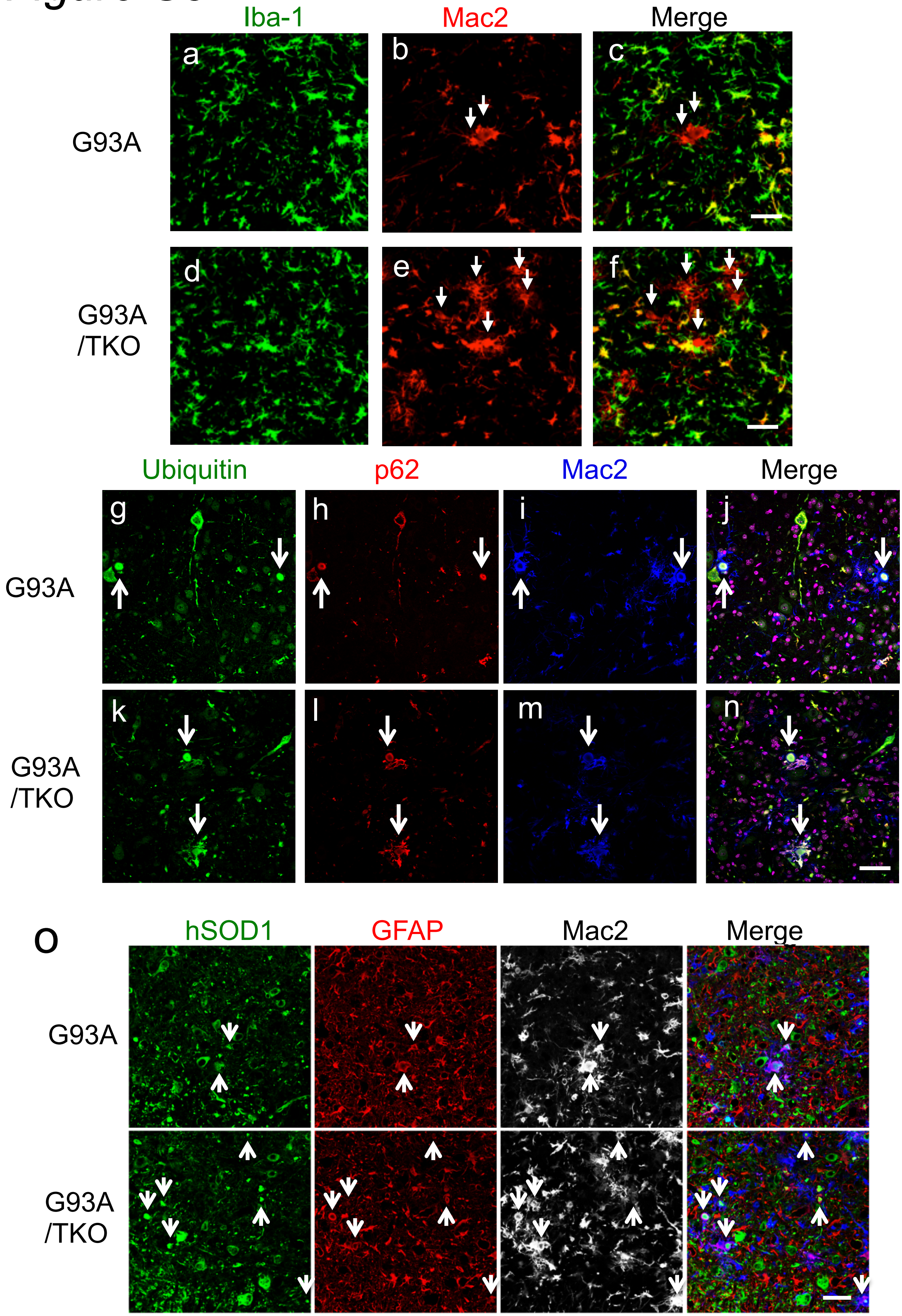
Iba-1
Mac2
Merge
a
b
c
G93A
d
e
f
G93A
/TKO
Ubiquitin
p62
Mac2
Merge
g
h
i
j
G93A
k
l
m
n
G93A
/TKO
O
hSOD1
GFAP
Mac2
Merge
G93A
G93A
/TKO

Figure S4

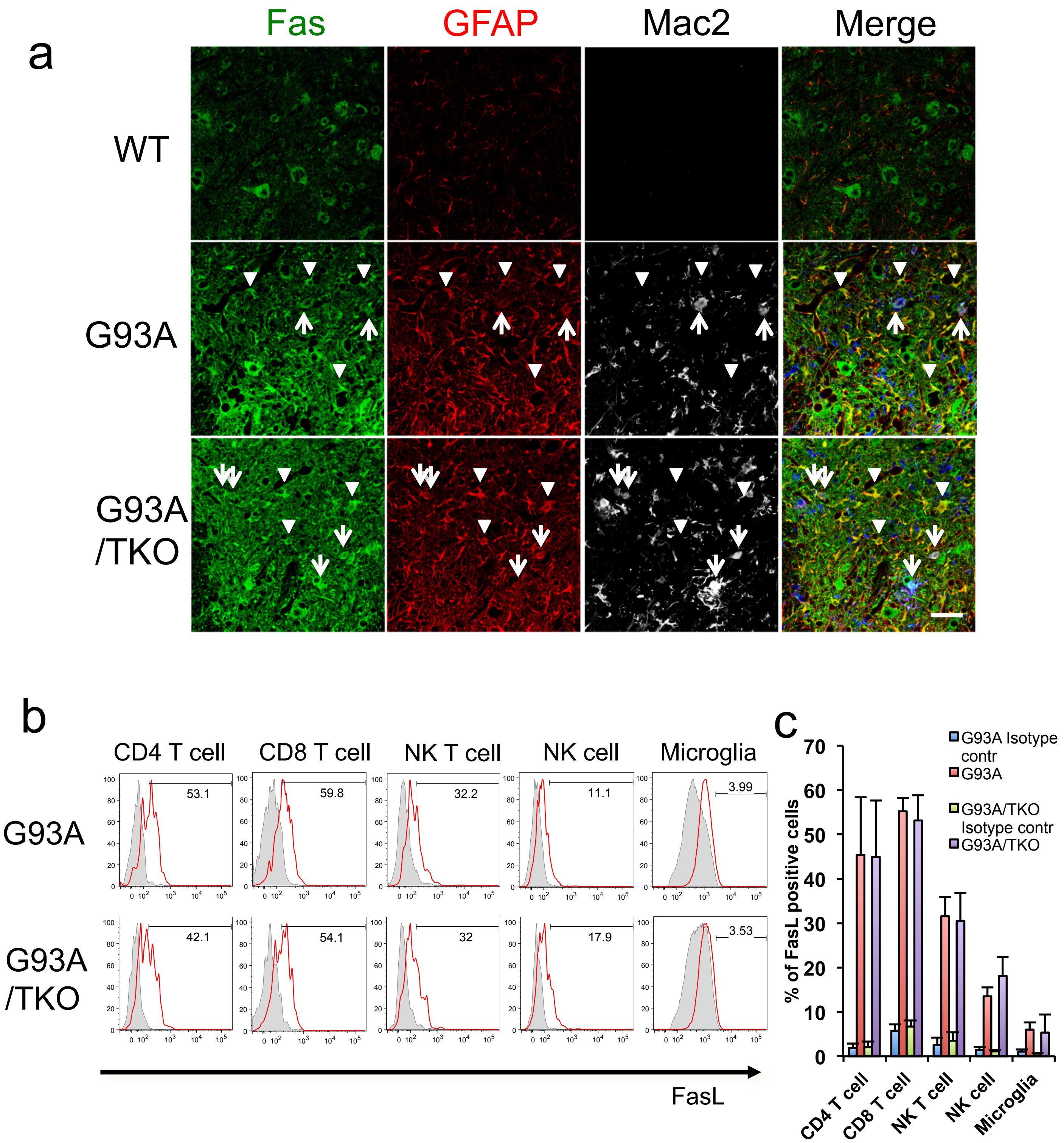
a
Fas
GFAP
Mac2
Merge
WT
G93A
G93A /TKO
b
CD4 T cell
CD8 T cell
NK T cell
NK cell
Microglia
G93A
G93A /TKO
53.1
59.8
32.2
11.1
3.99
42.1
54.1
32
17.9
3.53
FasL
c
G93A Isotype contr
G93A
G93A/TKO Isotype contr
G93A/TKO
% of FasL positive cells
CD4 T cell
CD8 T cell
NK T cell
NK cell
Microglia

Table S1

	Forward	Reverse
CCL5	GCTGCTTTGCCTACCTCTCC	TCGAGTGACAAACACGACTGC
CXCL10-1	GATGACGGGCCAGTGAGAA	GCTCGCAGGGATGATTTCAA
CXCL10-2	AGTCCTCGCTCAAGTGGCTGGGAT	AGTCCTCGCTCAAGTGGCTGGGAT
CCL2	TTAAAAACCTGGATCGGAACCAA	GCATTAGCTTCAGATTTACGGGT
CD14	GCACACTCACTCAACTTTTCCT	GCTGAGATCAGTCCTCTCTCG
CX3CR1	GAGTATGACGATTCTGCTGAGG	CAGACCGAACGTGAAGACGAG
GFAP	TGACCGCTTTGCTAGCTACATC	CCAGCGCCTTGTTTTGCT
IGF-1	CTGGACCAGAGACCCTTTG	CCTGTGGGCTTGTTGAAGTAAAA
GDNF	AGAGGGGCAAAAATCGGGG	CCGCTGCAATATCGAAAGATCA
BDNF	CCATAAGGACGCGGACTTGTA	TTTGCGGCATCCAGGTAATTT
TGF-β	GCAGTGGCTGAACCAAGGA	AGCAGTGAGCGCTGAATCG
TNF-α	CCCTCACACTCAGATCATCTTCT	GCTACGACGTGGGCTACAG
IFN-β	CGGACTTCAAGATCCCTATGGA	TGGCAAAGGCAGTGTAACTCTTC
IFN-γ	TGGCATAGATGTGGAAGAAAAGAG	TGCAGGATTTTCATGTCACCAT
ISG15	ATGAGGTCTTTCTGACGCAG	AGCAGCTCCTTGTCCTCCAT
IL-23	AAAATAATGTGCCCCGTATCCAG	GCTCCCCTTTGAAGATGTCAG
β−actin	TTGGCCTCACTGTCCACCTT	CGGACTCATCGTACTCCTGCTT
GAPDH	TGGCCTTCCGTGTTCCTAC	GAGTTGCTGTTGAAGTCGCA
